# Deciphering agronomic traits, biochemical components, and color in unique green-seeded fenugreek (*Trigonella foenum-graecum* L.) genotypes

**DOI:** 10.3389/fnut.2025.1542211

**Published:** 2025-02-27

**Authors:** Ravindra Singh, Ram Swaroop Meena, Sharda Choudhary, Narottam Kumar Meena, Ram Dayal Meena, Arvind Kumar Verma, Mahesh Kumar Mahatma, Ravi Yathendranaik, Shiv Lal, Pooja Kanwar Shekhawat, Vinay Bhardwaj

**Affiliations:** ICAR-National Research Centre on Seed Spices, Ajmer, India

**Keywords:** obesity, insulin-resistance, cancer, seed color, medicinal, harvest index, PCA

## Abstract

Fenugreek is a high-value legume known for its potential to enhance human health and combat a variety of diseases and metabolic disorders. This versatile crop has demonstrated promising therapeutic effects in managing obesity, diabetes, cancer, and poor metabolism conditions that have become major global health concerns. Despite the availability of multiple pharmaceutical remedies for these ailments in the market, often times the heavy chemical doses are accompanied by side effects on human body. To investigate the agronomic traits, medicinal potential, and color of fenugreek seeds, this study was conducted and identified fenugreek genotypes with green seed color (GSF1 to GSF10), which can prevent the progression of aforementioned diseases without the hassle of side effects. Ten unique green-seeded fenugreek (GSF) genotypes were compared with five released varieties (yellow-seeded fenugreek; YSF1 to YSF5) as check. The genotypes were assessed during rabi season for 3 consecutive years (2021–22 to 2023–24) in semi-arid Eastern Plain Zone of Rajasthan, India. The findings exhibited that agronomically GSF performed well, almost at par with the YSF. Harvest index (23.21 ± 0.37%) is higher in GSF with very marginal differences in other agronomic traits. The medicinal potential of the GSF showed that GSF6 has nearly 1.5 to 2 times higher insulinotropic 4-hydroxyisoleucine (0.90%) levels compared to the YSF genotypes. This unique non-protein branched amino acid is found in fenugreek seeds. GSF1 has a high concentration of chlorophyll (0.45 mg/100 g), GSF10 has low diosgenin and high 4-OHIle (261.80 mg/100 g and 0.85%, respectively), and GSF9 has low total soluble sugars (TSS; 3.50%). Oil content, phenols, and proteins were found to be higher in GSF making it preferable over YSF. The study further revealed that darkness of green color in the seed is directly related to its chlorophyll content and is directly associated with higher content of 4-OHIle and lower TSS. Among the studied genotypes, harvest index is higher in green-seeded genotypes with maximum seed yield (2473.74 Kg/ha) in genotype GSF8. The superior genotypes GSF1, GSF6, GSF8, GSF9, and GSF10 developed in the study hold potential for future breeding initiatives, aimed at boosting medicinal value, nutritional quality, and productivity.

## Introduction

Fenugreek (*Trigonella foenum-graecum* L.), from the Leguminosae family, is an economically and medicinally important crop with a chromosome number of 2n = 16 (1). Fenugreek is a well-known seed spices crop, cultivated all over the world (2), commercially grown in India, Pakistan, Iran, Egypt, Turkey, Middle East, and North Africa (3). Its commercial and medicinal benefits have made it a popular crop which is grown in almost all parts of the country since ages. Its leaves and seeds have been found to be effective in medicinal preparations. They are used as food for humans and as fodder for animals and improve soil health by augmenting the availability of nitrogen (4). Leaves of this plant are pinnate and long stalked compound toothed (5). Seeds are small, hard, and yellow to brownish yellow in color with smooth texture (3). Leaves and seeds are commonly used in ancient medicinal herbs containing minerals such as potassium, magnesium, calcium, zinc, manganese, copper and iron (6), vitamins (7), and *β*-carotene (5). The seed is typically yellow-colored endosperm with a bitter taste. It is rich in lipid lowering agent 4-hydroxyisoleucine (8, 9), diosgenin (2), chlorophyll (10), protein, flavonoids, carbohydrates, free amino acids, essential oil, seed oil (11–13), and other medicinally important compounds.

The WHO reports revealed that 422 million adults were suffering from diabetes worldwide in 2014 and accounted for 6.7 million deaths in 2021. It is predicted to rise further, reaching as much as 366 million by 2030 and 783 million by 2045 (14, 15). Diabetes mellitus is now approaching epidemic proportions (16). The same report revealed that cancer is the second disease causing an estimated 10 million deaths globally (17). Cancer is predicted to become the leading cause of diabetes-related deaths in older people with type 2 diabetes overtaking cardiovascular diseases (18). Both these diseases may further stimulate other conditions such as neuropathy, nephropathy, retinopathy, chronic kidney disease, skin complications (19), heart attack, stroke (20) obesity, PCOD, infertility, hair loss, and many more. Natural dietary sources and balanced lifestyle might play a key role in the treatment and even prevent these diseases. There are many natural dietary sources such as green vegetables, fresh fruits, dry fruits, nuts, and cereals for a healthy mind and body. Some key components such as 4-hydroxyisoleucine and diosgenin have potential to manage diabetes and tumor formation (21–23). Insulin secretagogue properties of 4-OHIle and anti-cancerous properties of diosgenin support its consumption for treatment of insulin resistance, cancer, diabetes, and obesity (24, 25).

Both 4-OHIle and diosgenin are naturally found in fenugreek seeds. Multiple reports are available claiming the medicinal potential of fenugreek seeds for antidiabetic, anti-cancer, gastric stimulation, and antibacterial (26–28) properties. Bakhtiar et al. (29) reported light brown, brown, and olive colored seeds in Iranian fenugreek genotypes and studied their phytochemical traits and antioxidant properties. Fenugreek seeds are rich in nutraceutical properties, have positive effects on digestive system (30), and act as an anti-cancer agent (31, 32) and an antioxidant (26, 33, 34) and good for heart health (35).

Fenugreek is very hardy and farmer loving crop. Farming this crop is considered less risky as opposed to other seed spice crops. The presence of antioxidants and medicinal values increases its importance and enhances the marketing of the crop which leads to monetary benefit to the cultivators. Variability in seed color might enhance quality and visual attractiveness for consumers. Despite decent potential of yellow-seeded fenugreek as medicinal crop, new genotypes/varieties with more 4-OHILe and diosgenin content find greater allure due to their distinguished green color. The objective of the present study is to investigate the variation in morphological characteristics and identify the superior high 4-OHILe and balanced diosgenin content by biochemical characterization as well as color categorization based on L*a*b* and chlorophyll content. The study also takes into account yield potential and capability for healthy and functional sustenance.

## Materials and methods

### Plant material, experimental site, and experimental design

**Figure 1:**
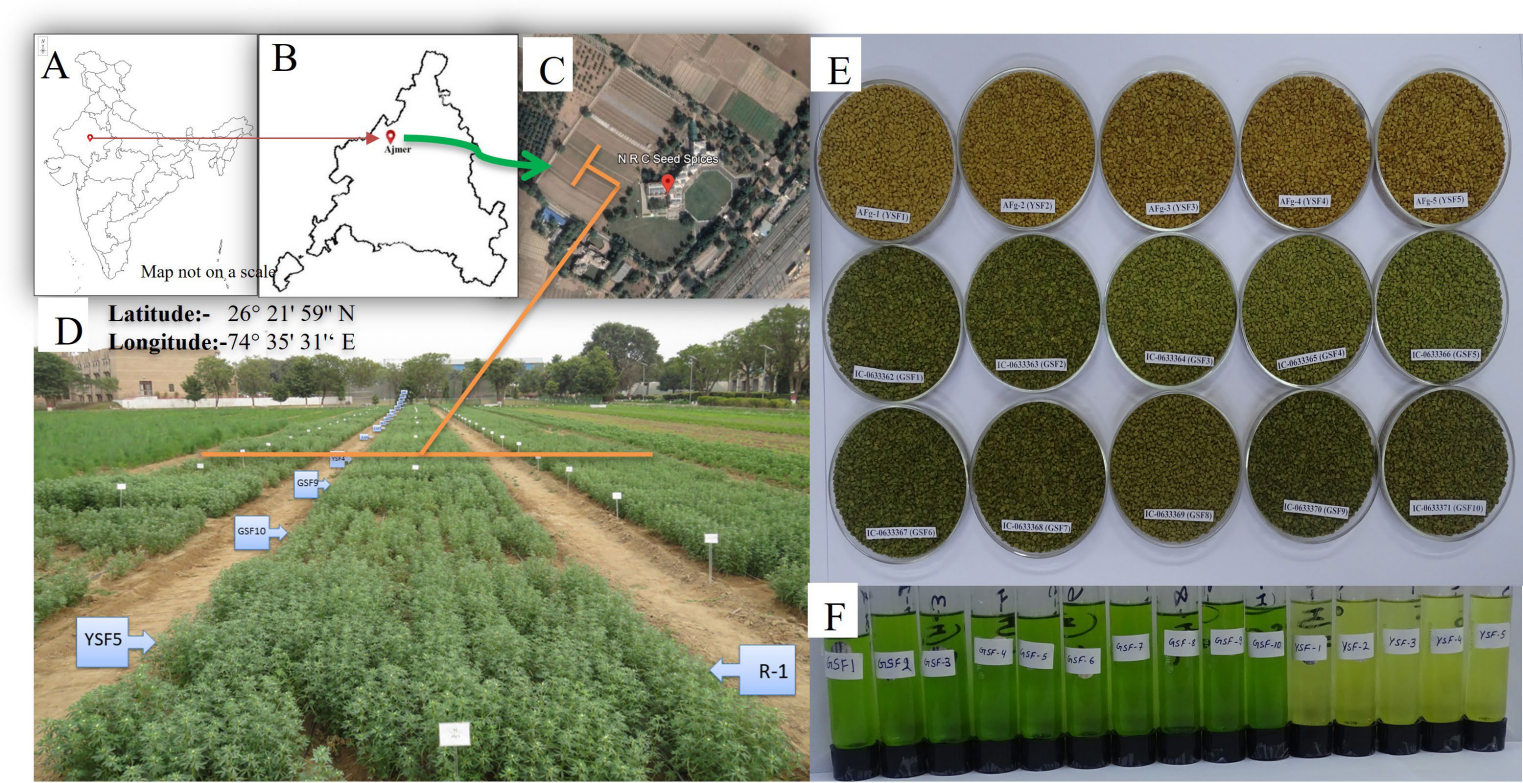
**(A–C)** Geo location of experimental site (India, Ajmer, ICAR-NRCSS), **(D)** Field view of experiment, **(E)** Seeds after harvesting and **(F)** Extract of seeds.

**Figure 2:**
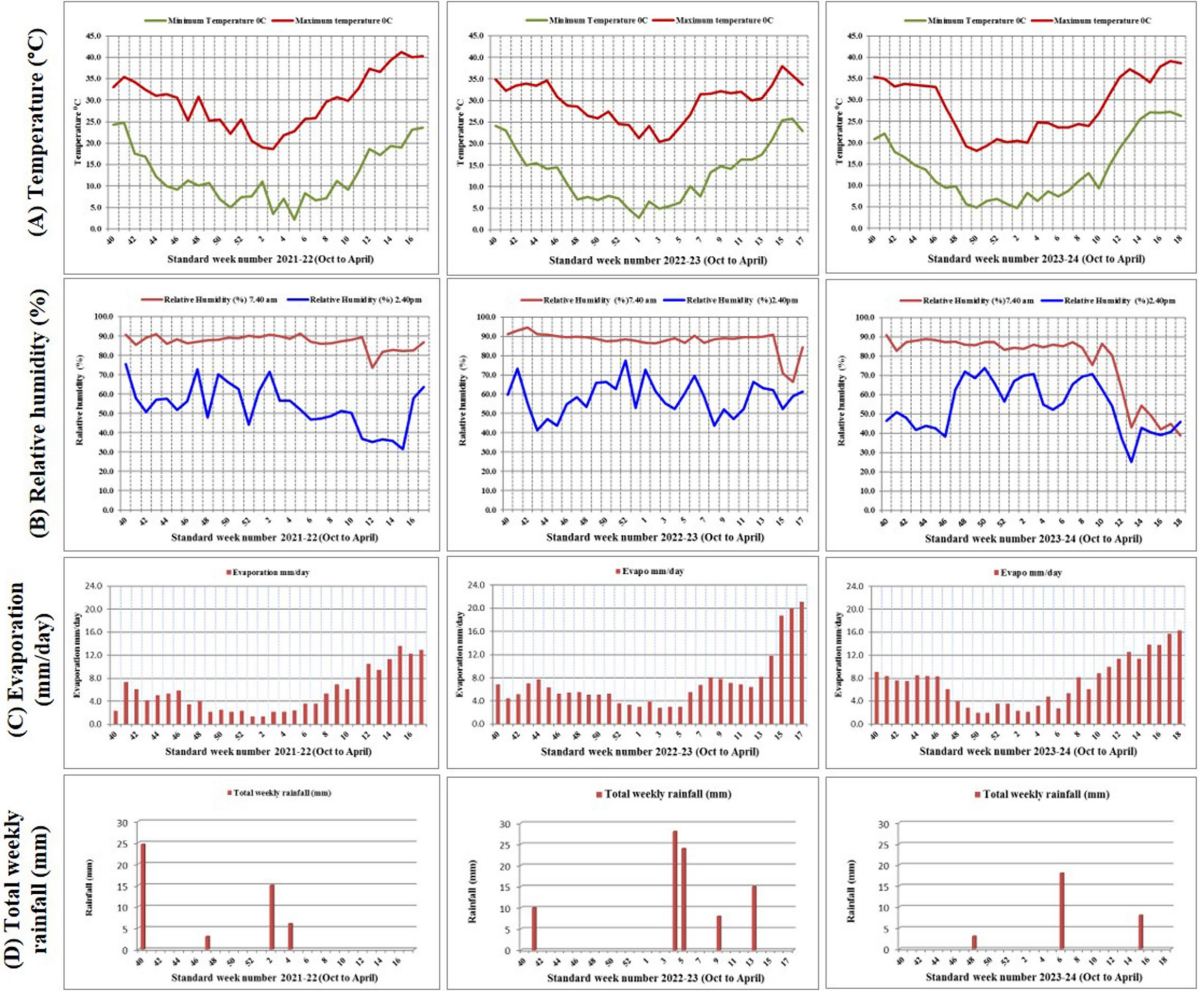
Meterological graph of 2021–22, 2022–23 and 2023–24 depicting **(A)** Temperature (°C) **(B)** Relative humidity (%) **(C)** Evaporation (mm/day) **(D)** Total weekly rainfall (mm).

Green-seeded fenugreek plants were collected from the Agro climatic Zone-IIA (Transitional Plain of Inland Drainage) of Rajasthan, India. Collected genotypes were maintained and multiplied at Indian Council of Agricultural Research - National Research Centre on Seed Spices (ICAR-NRCSS) field. After 3 years of field experiments, the pure green-seeded fenugreek germplasm lines with stable color character were selected. All the ten germplasm lines, identified for green seed color, were registered at Indian Council of Agricultural Research-National Bureau of Plant Genetic Resources (ICAR-NBPGR), New Delhi, India, for unique Indian Collection (IC) number *viz*: IC-0633362 (GSF1), IC-0633363 (GSF2), IC-0633364 (GSF3), IC-0633365 (GSF4), IC-0633366 (GSF5), IC-0633367 (GSF6), IC-0633368 (GSF7), IC-0633369 (GSF8), IC-0633370 (GSF9), and IC-0633371 (GSF10) (Supplementary Table S1). All the ten genotypes were evaluated for agro-morphological and medicinally important compounds. Total fifteen genotypes including five varieties *viz:* AFg-1 (YSF1), AFg-2 (YSF2), AFg-3 (YSF3), AFg-4 (YSF4), and AFg-5 (YSF5) as checks were planted in the field in Randomized Block Design (RBD), three replications with plot size 3×3 m^2^ with crop geometry 30×10 cm, during *rabi* season of 2021–22, 2022–23, and 2023–24. The experiment was conducted at research farm of ICAR-National Research Centre on Seed Spices (ICAR-NRCSS), Tabiji, Ajmer, situated at longitude 74° 35′ 31′ E and latitude 26° 21′ 59” N, at an altitude of 460.17 m above mean sea level. The region falls under agro climatic zone III a, “Semi-Arid Eastern Plain Zone” of Rajasthan (Figure 1). The field had a leveled topography and sandy loam soil texture. Mild winters and moderate summers with relatively high humidity from July to September are characteristics of this semi-arid and sub-tropical climatic zone. The mean annual rainfall is 550 mm, mostly received from southwest monsoon during the last week of June to September and the total rainfall during 2021–22, 2022–23 and 2023–24, i.e., the growing period of fenugreek was 24, 75, and 21 mm, respectively. The mean weekly meteorological observations recorded during the crop periods at the meteorological observatory of Research Farm, ICAR-NRCSS, Ajmer, are presented in Supplementary Table S2 and depicted in Figure 2. Data revealed that during growing crop cycle, maximum temperature ranged between 18.1°C to 39.8°C, 20.4°C to 34.6°C, and 18.1°C to 37.1°C, while minimum temperature ranged between 2.1°C to 20.5°C, 2.7°C to 20.9°C, and 4.7°C to 25.4°C during *rabi* 2021–22, 2022–23, and 2023–24, respectively. The mean daily maximum and minimum relative humidity varied between 43.1 and 90.7% during morning and 32.5 and 77.3% during afternoon across the years. Total annual rainfall during the years 2021–22, 2022–23, and 2023–24 was 624.7 mm, 915.3 mm, and 996.0 mm, respectively, in the last 2 years; it was extremely high over average annual rainfall. The soil of experimental field had a salinity of 0.83 ds/m, pH of 8.2, organic matter of 0.28%, and lime of 5.6%, with available nitrogen, phosphorus, and potassium as 155.7 kg/ha, 12.7 kg/ha, and 240 kg/ha, respectively.

### Observations of agronomic traits

The agronomic data recorded in the study included plant height (PH), primary branches (PB), secondary branches (SB), number of pods per plant (PPP), pod length (PL), secondary branches per plant (SPP), days to flowering (DF), maturity duration (MD), seed yield (SY), straw yield (StY), biological yield (BY), harvest index (HI), and test weight (TW). The plant height, primary branches, number of pods per plant, pod length, and number of secondary branches were recorded from randomly selected five plant from each plot, and other yield and yield attributes, *viz,* seed yield, straw yield, biological yield, harvest index, and test weight, were recorded after harvesting on plot basis. All the figures and tables generated are based on the pooled data analysis of three years.

### Biochemical compounds

Fully matured harvest of green-seeded genotypes and yellow-seeded genotypes from the year 2023–24 were used for analysis of biochemical components such as (4-OHIle 4-hydroxyisoleucine), diosgenin, chlorophyll, free amino acid (FAA), total soluble sugar (TSS), protein, and oil.

Chlorophyll contents of green- and yellow-seeded fenugreek were determined following the spectrophotometric assay described by Mazza and Oomah (36). Dry seed samples (1 g) were grinded and extracted in a 50 mL centrifuge tube and homogenized with 18 mL acetone: 1 mL NH_4_OH (0.1 N) solution for 30 min. Homogenates were stored in dark at 4°C for 2 min and then centrifuged at 5000 rpm. The supernatant was transferred to another test tube and read on a spectrophotometer (LAB INDIA Brand, Model no.3000+) at 700, 663, 645, and 626 nm. The reading taken at 700 nm was subtracted from each of the readings taken at 663, 645, and 626 nm. Total chlorophyll was calculated by summing the value of chlorophyll a and b after calculating them separately using the following formula:


$$\begin{array}{l} \mathrm{mg}/g\;\mathrm{Chlorophyll}\;a=14.18\;X\;OD\;663\times 2.91\;X\;OD\;645\\ \;–0.22\;X\;OD\;626 \end{array}$$

and


$$\begin{array}{l} m/g\;\mathrm{Chlorophyll}\;b=26.01\;X\;OD\;645–4.66\;X\;OD\;663\\ \;–0.36\;X\;OD\;626 \end{array}$$

A non-proteinogenic amino acid, 4-hydroxyisoleucine, from fenugreek seeds was extracted in 80% ethanol followed by 100% ethyl acetate. One gram of ground fenugreek seeds were taken, and 10 mL of 80.0% ethanol was added and filtrated. Extraction was repeated twice and pooled. Volume of the extract was reduced by evaporation in drybath (LABQUEST, model LBH010). Free amino acids were estimated by ninhydrin reagent. A standard curve was prepared with the use of leucine (0, 10, 20, 30, and 40 μg). For the estimation of 4-hydroxyisoleucine, amount of free amino acid was multiplied by 0.80 (37).

Saponins were extracted from fenugreek seed powder using 100% absolute ethanol (97) with continuous shaking in a shaker for 48 h. After extraction, solution was centrifuged for 20 min at 1000 rpm, and the supernatant was evaporated in drybath (LABQUEST, model LBH010) at 50°C. Dry residue was weighed and expressed as saponin content. Dry saponins were dissolved in 2 mL of 100% ethyl acetate in capped test tubes. To this, 1 mL of color-developing reagents consisted 0.5 mL of 0.5% (v/v) p-anisaldehyde and 0.5 mL of 50% H_2_SO_4_ was added. Both p-anisaldehyde and H_2_SO_4_ were prepared in ethyl acetate. The tubes were placed in a 60°C water bath for 10 min for color development; after that, 0.5 mL of distilled water was added to each tube.

A blank reagent was prepared similar way by taking 2 mL of ethyl acetate instead of samples. Ethyl acetate (100%) alone served as the control. Absorbance was measured at 430 nm on a LAB INDIA make spectrophotometer (Model no.3000+). A diosgenin calibration curve was prepared using standard solutions of diosgenin (10–80 μg/mL) prepared in 100% ethyl acetate. TSS were extracted in 80% ethanol and estimated by anthrone reagents. The intensity of color was read at 600 nm on spectrophotometer. A standard curve was prepared using 10 mg glucose per 100 ml distilled water (98).

Total amino acids were extracted in 80% ethanol and estimated by using ninhydrin reagent as described by Lee and Takahanshi (38). Total nitrogen content of the seeds was estimated by the method of Kjeldahl. Nitrogen content was multiplied by 6.25 factor and expressed as protein percent. Total oil from seeds of fenugreek was estimated by Soxhlet extraction method using n-hexane as solvent following the method of AOAC (39).

### Color analysis

The colorimetric analysis of the samples was conducted using a handheld digital colorimeter (CR-400 Chroma Meter, Konica Minolta Sensing Americas, Inc.). The instrument was calibrated using black and white tiles to assess the variations in their color properties, focusing on lightness (L*), chromaticity coordinates (a* and b*), hue angle (h*), chroma (C*), color differences (ΔE), greenness index (GI), and yellowness index (YI). The results revealed significant differences among the samples. ‘colordesigner.io’ (40) tools were used to identify the color name and shade.

### Statistical analysis

Statistical analysis was performed for all the traits observed, wherein mean values from three crop seasons *rabi* 2021–22, *rabi* 2022–23, and *rabi* 2023–24 with three replications were used. The descriptive statistics was performed for all observed traits using data analysis tool in MS Office Excel program. All the data were subjected to analysis of variance followed by mean comparison by *post-hoc* test. The means of green- and yellow-seeded fenugreek genotypes are presented as “Mean ± SE” and were compared using “Duncan’s multiple range test” (DMRT) in R studio statistical software with package “Agricolae.” Different lowercase letters indicate a significant difference, whereas mean values with the same lowercase letters are not significantly different (*p* < 0.05). Violin plots integrating boxplots for agronomical traits and two-way clustering heatmap were performed in SRplot (41). Line graph as well as radar for biochemical traits and seed color representation was performed in MS Office Excel. By utilizing mean of all the traits, correlation analysis, scree plot, variable principal component analysis, PCA biplot, and two-way clustering heat map were constructed. Pearson’s correlation analysis was performed by using *R* studio statistical software package “metan” to determine the relationship among traits. Principal component analysis and linear regression analysis were performed in General R based Analysis Platform Empowered by Statistics (*GRAPES 1.1.0*) (42).

## Results

### Agronomic traits

**Figure 3:**
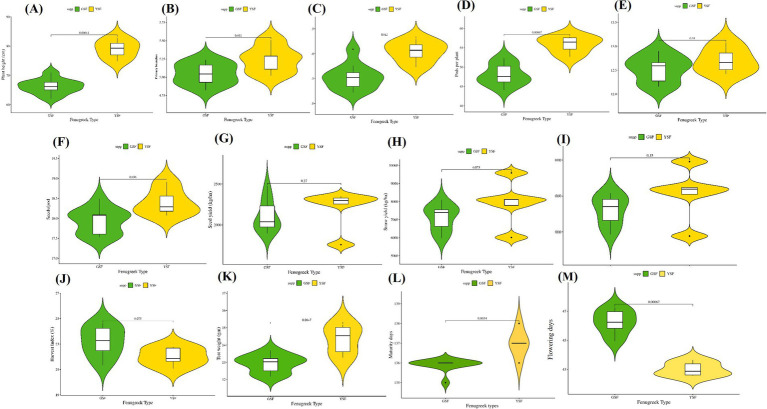
Comparative violin and boxplots of different morphological traits in green and yellow seeded fenugreek genotypes **(A)**. Plant height **(CM)**, **(B)**. Primary branches, **(C)**. Secondary branches, **(D)** Pods per plant, **(E)** Pod length (cm), **(F)** Seeds per pod, **(G)** Seed yield (Kg/ha), **(H)** Straw yield (Kg/ha), **(I)** Biological yield (Kg/ha), **(J)** Harvest Index (%), **(K)** Test weight (gm), **(L)** Maturity days, **(M)** Days to flowering.

Field experiment was carried out in a randomized block design for three winter seasons (*Rabi* 2021–22, *Rabi* 2022–23, and *Rabi* 2023–24). Significant differences were observed among the genotypes for all measured traits (*p* < 0.01, Table 1) suggesting great variations among fenugreek genotypes. Violin plots for agronomic traits are represented in Figure 3.

**Table 1:** Range, mean, standard error (SE), and coefficient of variation for different morphological traits in fenugreek.

2021–22						2022–23			2023–24			Pooled		
SN	Traits	Type	Mean ± SE	Range	CV	Mean ± SE	Range	CV	Mean ± SE	Range	CV	Mean ± SE	Range	CV
1	PH (cm)	GSF	74.39 ± 1.09	69.60–81.53	4.63	68.96 ± 1.60	61.93–78.07	7.32	55.34 ± 1.95	44.60–66.53	11.13	66.23 ± 0.81^b^	62.04–71.01	3.85
		YSF	77.79 ± 1.99	73.60–84.93	5.73	80.89 ± 2.62	71.47–86.80	7.24	78.32 ± 1.36	75.33–82.67	3.88	79 ± 1.41^a^	74.84–82.84	4
2	PB	GSF	4.38 ± 0.15	3.73–4.93	10.6	5.173 ± 0.12	4.68–5.93	7.14	5.59 ± 0.08	5.13–5.93	4.73	5.05 ± 0.05^b^	4.82–5.24	2.88
		YSF	4.47 ± 0.20	4.00–5.20	9.9	5.61 ± 0.04	5.47–5.73	1.76	5.65 ± 0.08	5.47–5.87	3.16	5.24 ± 0.08^a^	5.02–5.51	3.6
3	SB	GSF	7.42 ± 0.10	6.87–7.87	4.3	6.44 ± 0.15	5.53–7.20	7.56	5.74 ± 0.11	5.40–6.47	6.18	6.53 ± 0.08^b^	6.22–7.09	3.95
		YSF	7.68 ± 0.17	7.20–8.07	5.08	7.47 ± 0.23	6.73–8.13	6.8	5.97 ± 0.12	5.73–6.40	4.37	7.04 ± 0.1^a^	6.73–7.29	3.07
4	DF	GSF	45.54 ± 0.6	43–48	4.16	47.8 ± 0.13	47–48	0.88	45.4 ± 0.15	45–46	1.07	46.14 ± 0.27^a^	44.94–47.25	1.73
		YSF	42.05 ± 0.58	41–44	3.09	45.00	-	-	42.00	-	-	43.12 ± 0.24^b^	42.57–43.64	1.12
5	MD	GSF	141.85 ± 0.25	141–142.67	0.53	140.00 ± 0.29	138.67–140.33	0.63	126.00 ± 0.10	126	-	135.81 ± 0.06^b^	135.67–136.22	0.12
		YSF	141.42 ± 0.32	141–142.33	0.45	142.25 ± 0.25	142–143	0.35	126.83 ± 0.48	126–127.67	0.76	136.83 ± 0.21^a^	136.33–137.33	0.30
6	PPP	GSF	39.59 ± 0.72	35.80–43.40	5.74	58.37 ± 1.92	52.13–72.47	10.39	46.01 ± 1.56	40.73–55.13	10.71	47.99 ± 0.85^b^	44.11–52.31	5.59
		YSF	40.45 ± 1.50	37.00–45.53	8.28	78.65 ± 3.07	71.53–85.40	8.73	48.39 ± 3.01	40.87–58.73	13.89	55.83 ± 0.96^a^	52.69–57.78	3.85
7	PL (cm)	GSF	13.67 ± 0.16	12.93–14.40	3.63	12.02 ± 0.18	11.00–12.55	4.71	11.89 ± 0.01	11.59–12.47	2.35	12.52 ± 0.09^a^	12.16–12.9	2.15
		YSF	13.52 ± 0.12	13.27–13.87	1.92	12.50 ± 0.24	11.79–13.16	4.23	12.10 ± 0.14	11.75–12.53	2.67	12.71 ± 0.12^a^	12.42–13.08	2.09
8	SPP	GSF	17.34 ± 0.19	16.53–18.07	3.54	17.96 ± 0.12	17.20–18.47	2.07	18.49 ± 0.12	18.07–19.13	1.98	17.93 ± 0.1^b^	17.53–18.49	1.78
		YSF	17.85 ± 0.14	17.47–18.20	1.72	18.83 ± 0.39	17.87–20.27	4.66	18.52 ± 0.21	17.80–18.93	2.49	18.4 ± 0.15^a^	18.07–18.91	1.84
9	SY (Kg/ha)	GSF	2536.99 ± 70.36	2073.33–2870.85	8.77	2472.07 ± 121.37	1978.52–2967.41	15.53	1311.82 ± 51.50	962.59–1582.96	12.42	2106.96 ± 59.27^a^	1897.9–2473.74	8.9
		YSF	2253.62 ± 279.63	1152.22–2617.41	27.75	2896.59 ± 79.39	2676.67–3094.44	6.13	1443.33 ± 31.57	1337.78–1508.15	4.89	2197.85 ± 110.89^a^	1759.01–2349.88	11.28
10	StY (Kg/ha)	GSF	7840.66 ± 224.65	6296.30–8463.67	9.06	9091.38 ± 471.74	6407.80–11461.50	16.41	4382.26 ± 125.65	3585.56–4978.15	9.07	7104.77 ± 223.16^a^	5967.22–8096.67	9.93
		YSF	7172.41 ± 937.15	3481.04–8592.59	29.22	10678.52 ± 1052.04	8479.30–14681.57	22.03	5931.07 ± 216.40	5352.25–6385.59	8.16	7927.33 ± 570.79^a^	6012.89–9587.29	16.1
11	BY (Kg/ha)	GSF	10377.65 ± 288.43	8369.63–11334.52	8.79	11563.46 ± 534.81	8458.17–13757.80	14.63	5694.07 ± 163.65	4548.15–6296.30	9.09	9211.73 ± 260.67^a^	7865.13–10166.8	8.95
		YSF	9426.03 ± 1214.24	4633.26–11174.41	28.8	13575.11 ± 1068.63	11155.96–17576.75	17.6	7374.40 ± 189.18	6857.80–7802.63	5.74	10125.18 ± 668.19^a^	7771.91–11915.67	14.76
12	HI (%)	GSF	24.50 ± 0.26	23.32–25.91	3.32	22.02 ± 0.72	18.35–24.24	10.29	23.09 ± 0.51	20.59–25.22	6.92	23.21 ± 0.37^a^	21.32–24.65	5.03
		YSF	24.04 ± 0.39	23.14–24.98	3.64	22.23 ± 1.06	18.32–24.06	10.65	19.75 ± 0.94	17.50–22.20	10.67	22.01 ± 0.32^a^	21.06–22.78	3.29
13	TW (gm)	GSF	14.43 ± 0.34	11.97–15.83	7.47	12.53 ± 0.33	10.90–14.03	8.28	11.83 ± 0.05	11.53–12.03	1.33	12.93 ± 0.16^b^	12.18–13.7	3.92
		YSF	14.29 ± 0.51	13.37–16.07	8.02	15.55 ± 0.68	13.50–16.83	9.74	13.14 ± 0.53	11.70–14.80	8.96	14.33 ± 0.38^a^	13.27–15.18	5.95

Data are presented as the mean ± SE. Different lowercase letters indicate a significant difference (*p* < 0.05); mean values with the same lowercase letters are not significantly different according to Duncan’s multiple range test (DMRT; *p* < 0.05).

### Plant height

The plant height of the fenugreek genotypes was significantly affected over the growing years. Similarly, the GSF and YSF genotypes showed statistical differences when grown under the same field conditions in *rabi* 2021–22, 2022–23, and 2023–24. Plant height ranges from 69.60 cm to 81.53 cm in GSF genotypes and from 73.60 cm to 84.93 cm in YSF genotypes in 2021–22. In 2022–23, plant height ranges from 61.93 cm to 78.07 cm in GSF and 71.47 cm to 86.80 cm in YSF genotypes. Similarly in 2023–24, plant height ranged from 44.60 cm to 66.53 cm in GSF and 75.33 cm to 82.67 cm in YSF genotypes. Pooled data for all the three experimental years revealed that plant height ranged from 62.04 cm (GSF1) to 71.01 cm (GSF6) for green-seeded genotypes and for yellow-seeded genotypes it was from 74.84 cm (YSF1) to 82.84 cm (YSF2) (Table 1).

### Primary and secondary branches per plant

The primary branches varied from 3.73 to 4.93 in GSF and from 4.00 to 5.20 in YSF in 2021–22, from 4.68 to 5.93 in GSF and from 5.47 to 5.73 in YSF in 2022–23, whereas it varied from 5.13 to 5.93 in GSF and from 5.47 to 5.87 in YSF in 2023–24 with pooled value over 3 years ranging from 4.82 (GSF9) to 5.24 (GSF5) in GSF and 5.02 (YSF5) to 5.51 (YSF3) in YSF genotypes. Both primary and secondary branches were comparable in GSF and YSF genotypes. Secondary branches varied from 6.87 to 7.87 in GSF and 7.20 to 8.07 in YSF during 2021–22, from 5.53 to 7.20 in GSF and 6.73 to 8.13 in YSF during 2022–23, and from 5.40 to 6.47 in GSF and 5.73 to 6.40 in YSF during 2023–24 with pooled value ranging from 6.22 (GSF3) to 7.09 (GSF7) in GSF and 6.73 (YSF5) to 7.29 (YSF1) in YSF genotypes (Table 1).

### Pod-related traits

Number of pods/plant ranged from 35.80 to 43.40 in GSF and 37 to 45.53 in YSF in 2021–22, from 52.13 to 72.47 in GSF and 71.53 to 85.40 in YSF in 2022–23, and from 40.73 to 55.13 in GSF and 40.87 to 58.73 in YSF in 2023–24 with pooled value of all the 3 years ranging from 44.11 (GSF10) to 52.31 (GSF7) in GSF and 52.69 (YSF3) to 57.78 (YSF4) in YSF (Table 1).

Pod length ranged from 12.93 cm to 14.40 cm for GSF genotypes and 13.27 cm to 13.87 cm for YSF genotypes in 2021–22, 11.00 cm to 12.55 cm for GSF and 11.79 cm to 13.16 cm for YSF in 2022–23, and 11.59 to 12.47 cm for GSF and 11.75 to 12.53 cm for YSF in 2023–24 with pooled value ranging from 12.1 6 cm (GSF8) to 12.90 cm (GSF7) for GSF and from 12.42 cm (YSF4) to 13.08 cm (YSF3) for YSF (Table 1).

Seeds per pod were comparable for both GSF and YSF genotypes over all three *rabi* seasons 2021–22, 2022–23, and 2023–24. Generally, it ranged from 17.53 (GSF5) to 18.49 (GSF9) in GSF and 18.07 (YSF1) to 18.91 (YSF5) in YSF (Table 1).

### Days to flowering

Flowering days ranged from 43 to 48 days for GSF (45.54 days) and 41 to 44 days for YSF (42.05 days) in 2021–22, from 47 to 48 days for GSF and up to 45 days for YSF in 2022–23, and from 45 to 46 days for GSF and 42 days for YSF in 2023–24 (Table 1).

### Maturity days

Maturity days ranged from 141 to 142.67 days for GSF (141.85 days) and from 141 to 142.33 days for YSF (141.42 days) in 2021–22, 138.67 to 140.33 days for GSF (140 days) and from 142 to 143 days for YSF (142.25 days) in 2022–23, and 126 days for GSF and from 126 to 127.67 days for YSF (126.83 days) in 2023–24, with pooled value ranging from 135.67 to 136.22 days for GSF (135.81 days) and 136.33 to 137.33 days for YSF (136.83 days) (Table 1).

### Seed yield

Seed yield showed significant differences in the *rabi* periods of (2021–22, 2022–23, and 2023–24) among the fenugreek genotypes grown under normal condition. Pooled data revealed that the highest seed yield was obtained from the genotypes GSF8 (2473.74 Kg/ha), followed by YSF1 (2349.88 kg ha-^1^), YSF4 (2328.38 kg ha-^1^), and GSF4 (2316.91 kg/ha) among all genotypes studied (Table 1).

### Straw yield

Straw yield ranged from 6296.30 kg ha-^1^ to 8463.67 kg ha-^1^ for GSF genotypes and 3481.04 kg ha-^1^ to 8592.59 kg ha-^1^ for YSF genotypes in 2021–22, 6407.80 kg ha-^1^ to 11461.50 kg ha-^1^ for GSF genotypes and from 8479.30 kg ha-^1^ to 14681.57 kg ha-^1^ for YSF in 2022–23, and 3585.56 kg ha-^1^to 4978.15 kg ha-^1^for GSF and 5352.25 kg ha-^1^to 6385.59 kg ha-^1^for YSF in 2023–24 with pooled value of 5967.22 kg ha-^1^(GSF10) to 8096.67 kg ha-^1^(GSF2) for GSF and 6012.89 kg ha-^1^(YSF3) to 9587.29 kg ha-^1^(YSF4) for YSF. Yellow-seeded fenugreek genotypes are the better performing genotype for straw yield (Table 1).

### Biological yield

Biological yield of fenugreek genotypes can vary significantly, depending on the genotypes and the environmental conditions. Biological yield ranged from 8369.63 kg ha-^1^ to 11334.52 kg ha-^1^a for GSF genotypes and 4633.26 kg ha-^1^ to 11174.41 kg ha-^1^ for YSF genotypes in 2021–22, 8458.17 kg ha-^1^ to 13757.80 kg ha-^1^ for GSF and 11155.96 kg ha-^1^ to 17576.75 kg ha-^1^ for YSF in 2022–23, and 4548.15 kg ha-^1^ to 6296.30 kg ha-^1^ for GSF and 6857.80 kg ha-^1^ to 7802.63 kg ha-^1^ for YSF in 2023–24 with pooled value ranging from 7865.13 kg ha-^1^ to 10166.80 kg ha-^1^ in GSF and from 7771.91 kg ha-^1^ to 11915.67 kg ha-^1^ in YSF. The genotypes with highest biological yield are YSF4 (11915.67 kg ha-^1^), YSF1 (10473.54 kg ha-^1^), and YSF2 (10376.29 kg ha-^1^) followed by green-seeded genotypes GSF2 (10166.80 kg ha-^1^) and GSF8 (10145.83 kg ha-^1^) (Table 1).

### Harvest index

Harvesting index was comparable for both GSF and YSF genotypes over all three seasons 2021–22, 2022–23, and 2023–24. In our study, it ranged from 23.32 to 25.91% for GSF genotypes and from 23.14 to 24.98 for YSF in 2021–22, from 18.35 to 24.24% for GSF and 18.32 to 24.06% for YSF in 2022–23, and from 20.59 to 25.22% for GSF and 17.50 to 22.20% for YSF with pooled value of 21.32% (GSF1) to 24.65% (GSF8) for GSF and 21.06% (YSF4) to 22.78% (YSF5) for YSF (22.01%) (Table 1).

### Test weight

Test weight (1,000 seed weight) depends on the genotype and other environmental factors. The test weight ranged from 11.97 g to 15.83 g for GSF and from 13.37 g to 16.07 g for YSF in 2021–22, from 10.90 g to 14.03 g for GSF and from 13.50 g to 16.83 g for YSF in 2022–23, and from 11.53 g to 12.03 g for GSF and 11.70 g to 14.80 g for YSF in the third year of experiment with pooled value ranging from 12.18 g (GSF5) to 13.70 g (GSF3) for GSF (12.93 g) and 13.27 g to 15.18 g for YSF (14.33 g) (Table 1).

### Biochemical analysis

#### Total chlorophyll content

In our study, chlorophyll content ranged from 0.31 mg/100 g (GSF9) to 0.45 mg/100 g (GSF1) with mean value of 0.36 mg/100 g in GSF genotypes and 0.02 to 0.04 mg/100 g with mean value of 0.03 mg/100 g in YSF genotypes (Table 2).

**Table 2:** Biochemical characterization of GSF and YSF fenugreek genotypes.

SN	Fenugreek type	Genotypes	Total Chl (mg/100 g)	4-OHIle (%)	Diosgenin (mg/100 g)	FAA (mg/100 g)	TSS (%)	Total phenol (mg/100 g)	Oil (%)	Protein (%)
1	Green-seeded fenugreek	GSF1	0.45	0.81	382.28	1016.79	5.52	21.22	3.74	21.67
		GSF2	0.36	0.75	412.55	931.02	5.15	41.55	3.53	15.69
		GSF3	0.33	0.84	338.88	1048.95	4.05	42.59	3.45	19.28
		GSF4	0.38	0.70	326.79	861.57	3.9	26.37	3.13	18.22
		GSF5	0.35	0.75	366.72	937.04	4.05	31.36	3.39	18.03
		GSF6	0.35	0.90	295.05	1124.76	5.6	55.94	3.51	20.55
		GSF7	0.33	0.88	370.51	1094.87	4.3	35.12	3.37	19.89
		GSF8	0.35	0.77	413.09	962.24	3.8	21.58	3.66	17.96
		GSF9	0.31	0.81	388.35	1016.73	3.5	38.98	3.71	18.57
		GSF10	0.40	0.85	261.84	1057.14	4.55	18.17	3.53	18.44
		Mean	0.36 ± 0.01^a^	0.81 ± 0.02^a^	355.61 ± 15.72^a^	1005.11 ± 25.79^a^	4.44 ± 0.23^b^	33.29 ± 3.75^b^	3.5 ± 0.06^b^	18.83 ± 0.52^b^
		Range	0.31–0.45	0.7–0.9	261.84–413.09	861.57–1124.76	3.5–5.6	18.17–55.94	3.13–3.74	15.69–21.67
2	Yellow-seeded fenugreek	YSF1	0.02	0.44	261.44	554.02	6.39	113.33	4.3	20.58
		YSF2	0.04	0.48	508.1	606.39	6.23	116.21	4.24	20.42
		YSF3	0.02	0.58	289.9	722.1	5.79	116.31	4.1	24.26
		YSF4	0.04	0.73	319.55	911.99	5.77	96.78	3.97	20.83
		YSF5	0.02	0.5	452.74	633.49	6.04	8.84	4.48	21.15
		Mean	0.03 ± 0.01^b^	0.55 ± 0.05^b^	366.34 ± 48.27^a^	685.6 ± 62.8^b^	6.04 ± 0.12^a^	90.29 ± 20.68^a^	4.22 ± 0.09^a^	21.45 ± 0.71^a^
		Range	0.02–0.04	0.44–0.73	261.44–508.1	554.02–911.99	5.77–6.39	8.84–116.31	3.97–4.48	20.42–24.26

Data are presented as the mean ± SE. Different lowercase letters indicate a significant difference (*P* < 0.05); mean values with the same lowercase letters are not significantly different according to Duncan’s multiple range test (DMRT; *p* < 0.05).

### 4-hydroxyisoleucine (%)

**Figure 4:**
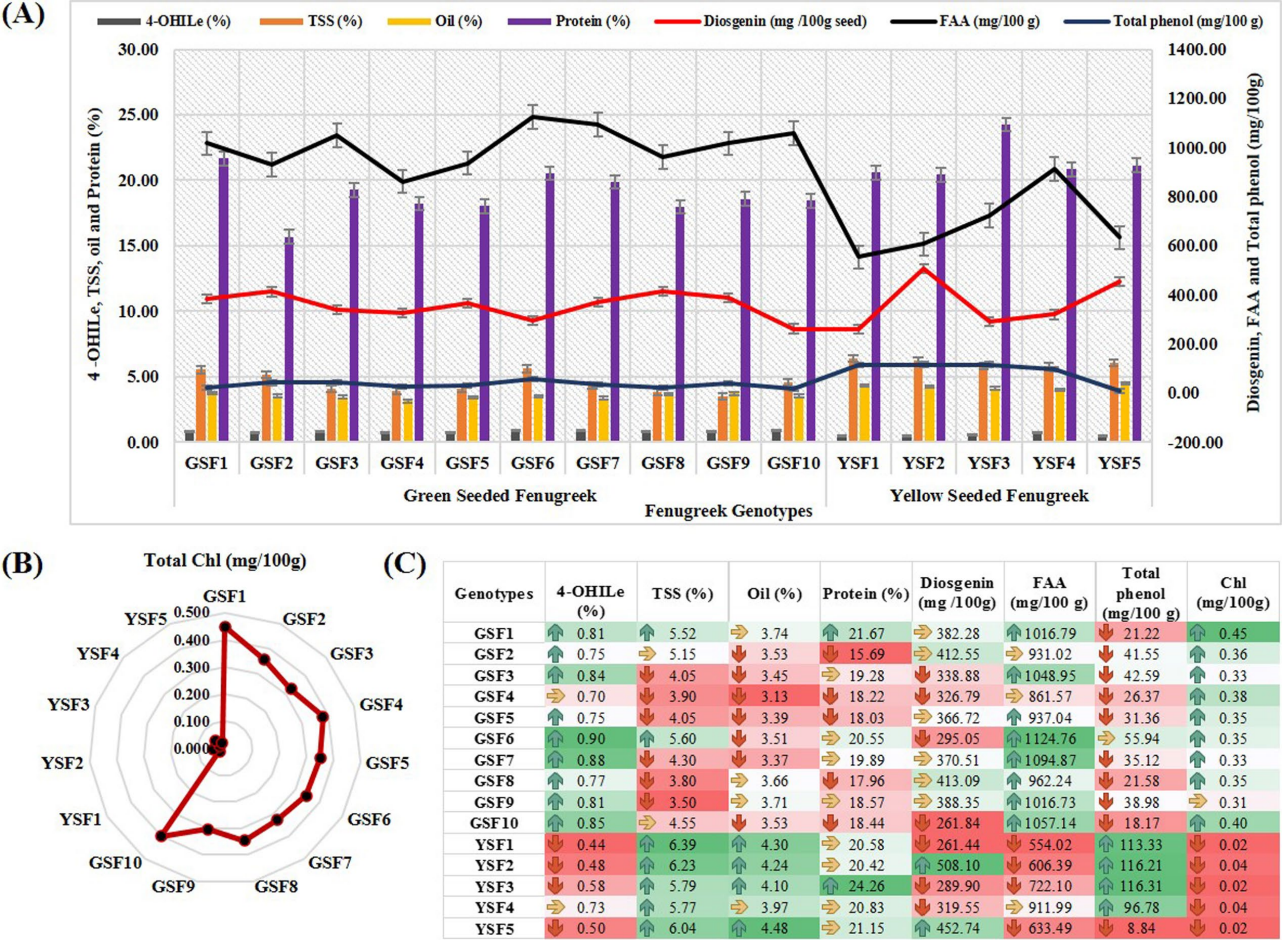
**(A)** Comparison of medicinal compounds, **(B)** Total Chl (mg/100 g) contents and **(C)** Comparative value representation for medicinal compound and chlorophyll content. [4-OHIle (4-Hydroxyisoleucine), TSS (Total soluble sugar), FAA (Free fatty acid), Chl (Chlorophyll content)].

The most abundant free amino acid in fenugreek, 4-hydroxyisoleucine (branched-chain amino acid), is found in the seed endosperm (43). Among the fenugreek genotypes studied, GSF1, GSF3, GSF6, GSF7, GSF9, and GSF10 exhibited the highest concentrations, measuring 0.81, 0.84, 0.90, 0.88, 0.81, and 0.85%, respectively, with mean value of 0.81% in green-seeded subgroup of fenugreek genotypes that is almost 1.5 times higher than yellow-seeded fenugreek genotypes with 0.44 to 0.73% (Table 2 and Figure 4).

### Total soluble sugar (%)

The present study revealed that total soluble sugar content in green-seeded fenugreek genotypes varied from 3.5% (GSF9) to 5.6% (GSF6) with mean value of 4.44%, i.e., much lower than (almost half) yellow-seeded fenugreek genotypes that contained 5.77% (YSF4) to 6.39% (YSF1) with mean value of 6.04% total soluble sugar (Table 2).

### Diosgenin (mg/100gm)

Diosgenin ranged from 261.84 mg/100 g (GSF10) to 413.09 mg/100 g (GSF8) with mean value of 355.61 mg/100 g in green-seeded fenugreek genotypes and 261.44 mg/100 g (YSF1) to 508.10 mg/100 g (YSF2) with mean value of 366.34 mg/100 g in yellow-seeded fenugreek genotypes (Table 2).

### Free amino acid (mg/100gm)

In our investigation study, FAA ranged from 861.57 mg/100 g to 1124.76 mg/100 g with average value of 1005.11 mg/100 g with higher content of 1124.76 mg/100 g in GSF6, 1057.14 mg/100 g in GSF10, and 1094.87 mg/100 g in GSF7. In case of yellow-seeded fenugreek, it ranged from 554.02 to 911.99 mg/100 g with mean value of 685.60 mg/100 g having higher content in YSF4 (911 mg/100 g) and 722.10 mg/100 g in YSF3 (Table 2).

### Protein content (%)

Protein content ranged from 15.69% (GSF2) to 21.67% (GSF1) with mean value of 18.83% in green-seeded fenugreek genotypes, whereas it ranged from 20.42% (YSF2) to 24.26% (YSF3) with mean value of 21.45% in yellow-seeded fenugreek genotypes (Table 2).

### Total phenol content (mg/100 g)

Total phenol content in this investigation study ranged from 18.17 (GSF10) to 55.94 (GSF6) mg/100 g with mean value of 33.29 mg/100 g in green-seeded fenugreek genotypes and from 8.84 (YSF5) to 116.31 (YSF3) with mean value of 90.29 mg/100 g (Table 2).

### Oil content (%)

Oil content in green-seeded fenugreek genotypes varied from 3.13% (GSF4) to 3.74% (GSF1) with mean value of 3.50% results consistent with yellow-seeded fenugreek genotypes (Table 2).

### Colorimetric characterization

#### Lightness (L*)

**Figure 5:**
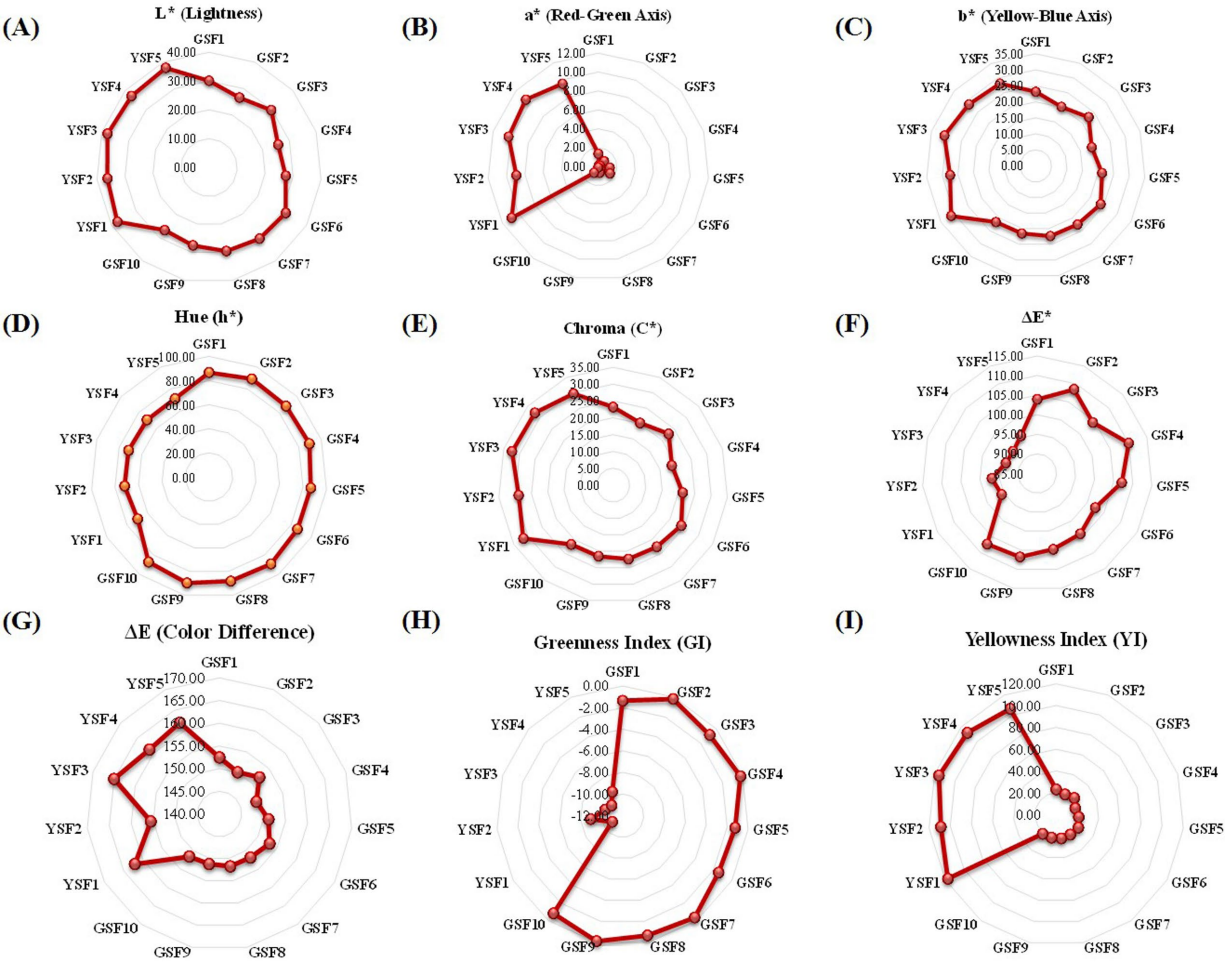
Radar graph representing the mean score of green and yellow seeded fenugreek genotypes **(A)** L* (Lightness) **(B)** a* (Red-Green Axis) **(C)** b* (Yellow-Blue Axis) **(D)** h* (Hue) **(E)** C* (Chroma) **(F)** ΔΕ True green **(G)** ΔΕ True yellow **(H)** (Greeness Index) **(I)** YI (Yellowness Index).

Lightness (L*), i.e., color brightness, ranged from 25.78 to 31.45 with mean value of 28.55 in GSF genotypes and from 36.31 to 37.93 with mean value of 37.34 in YSF genotypes. Colorimetric characterization values and respective graphs are represented in Figure 5 and Table 3.

**Table 3:** Colorimetric characterization of green- and yellow-seeded fenugreek genotypes.

SN	Type	sample	L*	a*	b*	h*	Chroma (C*)	ΔE from True green	Greenness Index (GI)	ΔE* (True yellow)	Yellowness Index (YI)	Color shade code	Color shade name
1	Green-seeded fenugreek	GSF1	30.08	1.36	23.13	86.66	23.18	152.45	−1.36	103.92	23.13	#564724	Dark olive green
		GSF2	26.56	0.18	20.18	89.07	20.21	150.07	−0.18	108.48	20.18	#493e20	Dark olive green
		GSF3	29.74	0.85	22.74	87.84	22.77	152.10	−0.85	104.43	22.74	#524522	Dark olive green
		GSF4	25.78	0.21	18.78	89.45	18.80	148.77	−0.21	110.03	18.78	#473c20	Dark slate gray
		GSF5	27.38	1.23	21.27	86.69	21.31	151.12	−1.23	107.13	21.27	#4d3f20	Dim gray
		GSF6	31.45	1.45	23.91	86.54	23.95	153.05	−1.45	102.43	23.91	#584824	Dark olive green
		GSF7	30.48	0.34	22.59	89.18	22.61	151.85	−0.34	104.05	22.59	#584724	Dark olive green
		GSF8	29.62	0.66	22.39	88.30	22.40	151.77	−0.66	104.78	22.39	#524423	Dark olive green
		GSF9	27.59	0.11	21.54	89.90	21.56	151.25	−0.11	106.78	21.54	#4c4020	Dark olive green
		GSF10	26.81	0.82	21.65	87.15	21.73	151.60	−0.82	107.25	21.65	#463e1e	Dark olive green
		Mean	28.55 ± 0.61^b^	0.72 ± 0.16^b^	21.82 ± 0.47^b^	88.08 ± 0.4^a^	21.85 ± 0.48^b^	151.4 ± 0.39^b^	−0.72 ± 0.16^a^	105.93 ± 0.75^a^	21.82 ± 0.47^b^		
		Range	25.78–31.45	0.11–1.45	18.78–23.91	86.54–89.9	18.8–23.95	148.77–153.05	−1.45 – −0.11	102.43–110.03	18.78–23.91		
2	Yellow-seeded fenugreek	YSF1	37.59	10.86	31.23	69.72	31.60	162.05	−10.86	95.72	117.51	#765125	Saddle brown
		YSF2	36.31	8.94	27.52	71.91	28.95	155.60	−8.94	96.92	108.88	#6f4f29	Saddle brown
		YSF3	37.93	10.24	30.70	71.67	32.40	165.01	−10.24	93.63	115.79	#765227	Saddle brown
		YSF4	37.04	10.59	28.72	70.76	32.09	161.24	−10.59	93.49	112.26	#745029	Saddle brown
		YSF5	37.84	9.58	28.14	71.09	29.73	161.99	−9.58	95.51	106.21	#74522b	Saddle brown
		Mean	37.34 ± 0.3^a^	10.04 ± 0.35^a^	29.26 ± 0.72^a^	71.03 ± 0.39^b^	30.95 ± 0.68^a^	161.18 ± 1.54^a^	−10.04 ± 0.35^b^	95.05 ± 0.66^b^	112.13 ± 2.1^a^		
		Range	36.31–37.93	8.94–10.86	27.52–31.23	69.72–71.91	28.95–32.4	155.6–165.01	−10.86 – −8.94	93.49–96.92	106.21–117.51		

Data are presented as the mean ± SE. Different lowercase letters indicate a significant difference (*P* < 0.05); mean values with the same lowercase letters are not significantly different according to Duncan’s multiple range test (DMRT; *P* < 0.05).

#### Red green Axis (a*)

Red green axis (a*) component of colorimetric analysis represented positive values for all genotypes that ranges from 0.11 to 1.45 in green-seeded fenugreek and 8.94 to 10.86 in yellow-seeded fenugreek genotypes (Table 3).

#### Yellow blue Axis (b*)

Color coordinate study of yellow blue axis indicated that all genotypes had positive value that ranged from 18.78 to 23.91 with mean value of 21.82 in GSF and 27.52 to 31.23 with mean value of 29.26 in YSF indicating their shift toward yellowness (Table 3).

#### Hue (h*)

Hue in the present investigation ranged from 86.54° to 89.90° with mean value of 88.08° in GSF and 69.72° to 71.91° with mean value of 71.03° in YSF fenugreek genotypes (Table 3).

#### Chroma (C*)

Chroma ranged from 18.80 to 23.95 with 21.85 in GSF and 28.95 to 32.40 with mean value of 30.95 in YSF genotypes. YSF3 (32.40), YSF4 (32.09), YSF1 (31.60), and YSF5 (29.73) exhibited more saturated vividness compared to the GSF genotypes (Table 3).

#### Color difference (ΔE*)

ΔE* value ranged from 148.77 to 153.05 with average value of 151.40 in GSF and 155.60 to 165.01 with average value of 161.18 in YSF genotypes indicating varying degrees of deviation from a green reference, with all samples showing significant differences (Table 3).

### Greenness index (GI)

Greenness index ranged from −1.45 to −0.11 with average value of −0.72 in GSF subgroup and − 10.86 to −8.94 with mean value of −10.04 in YSF subgroup of fenugreek genotypes. All GSF genotypes represented higher GI index indicating more greenness as compared to YSF subgroup of fenugreek genotypes (Table 3).

### Yellowness index (YI)

Yellowness index ranged from 18.78 to 23.91 with mean value of 21.82 in GSF and from 106.21 to 117.51 with mean value of 112.13 in YSF subgroup of fenugreek genotypes. YSF subgroup had maximum value indicating more yellowness of their genotypes among all fenugreek genotypes (Table 3).

### Color shade name and shade cade

Two types of genotypes were used in study: ten with green seed color (GSF) and five with yellow seed color (YSF). Green-seeded fenugreek genotypes are having three different shades, GSF1, GSF2, GSF3, GSF6, GSF7, GSF8, GSF9, and GSF10 are dark olive green, GSF4 is dark slate gray, and GSF5 is dim gray with different shade codes, whereas all the five YSF genotypes are saddle brown in color with different shade codes (Table 3).

### Principal component analysis

**Figure 6:**
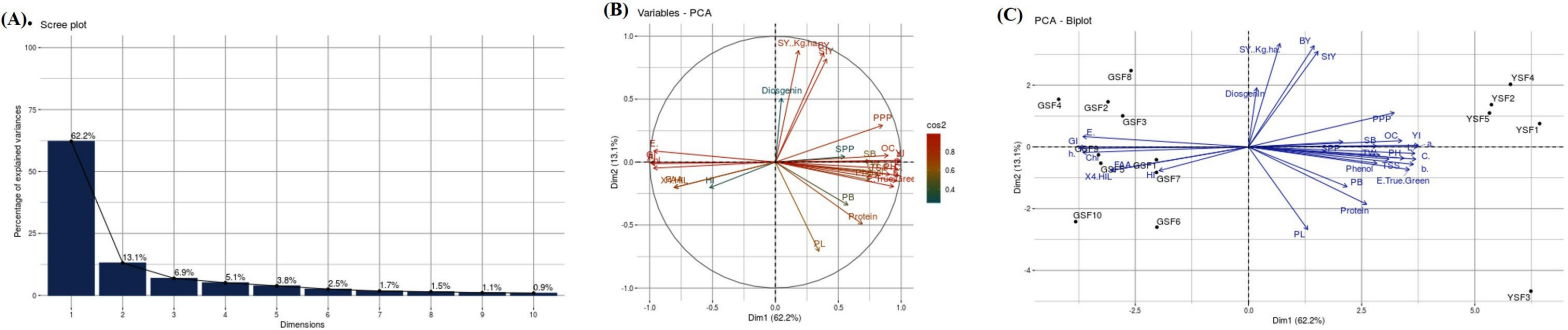
**(A)** Scree plot, **(B)** Variable PCA and **(C)** PCA biplot of Agronomic, Biochemical and color attributes representing variability Plant height (PH), primary branches (PB), secondary branches (SB), number of pods per plant (PPP), pod length (PL), secondary branches per plant (SPP), days to flowering (DF), maturity duration (MD), seed yield (SY), straw yield (StY), biological yield (BY), harvest index (HI) and test weight (TW), 4-hydroxyisoleucine (4-OHIle), Chlorophyll (Chl), Free amino acids (FAA), Total soluble sugars (TSS), Green Seeded Fenugreek (GSF) and Yellow Seeded Fenugreek (YSF).

PCA was conducted to dissect the variation patterns in fenugreek genotypes for all the studied traits. A scree plot, i.e., graphical approach (Figure 6A) was also created to represent the variability of each component. The PCA results for the GSF and YSF revealed that the total variation was dissected into 15 principal components (PCs). Out of 15 PCs, only 5 PCs presented more than 1.0 eigenvalue, whereas remaining PCs did not showcase significant variations. Individually, PC-1 had 62.18% of variance, whereas PC-2 and PC-3 illuminated 13.10 and 6.86% of the variability, respectively. Cumulatively, these three PCs described 82.14% of total variability among the attributes. PC-1 exhibited positive loading with 21 traits with maximum contribution delivered by yellowness index (0.228), red green axis (0.227), chroma (0.223), lightness (0.221), yellow blue axis (0.22), color difference (0.216), plant height (0.209), oil content (0.204), and pods per plant (0.195). In the case of PC-2, positive factor loadings were observed for 16 traits with maximum contribution delivered by pod length (0.37), protein content (0.25), primary branches per plant (0.162), harvest index (0.12), and 4-OHIle (0.103). To investigate the interaction between genotypes and traits, a genotype by trait biplot was created. The first two PC-1 and PC-2 accounted for 75.27% of the total variability and were used for the construction of a genotype by trait biplot. In the biplot, along the x-axis, the PC-1 score was plotted, and along the y-axis, the PC-2 score was plotted along with all fenugreek genotypes.

To understand the interrelationship of all investigated traits, a vector line for all traits was drawn from the origin. Through this approach, we were able to group genotypes according to their relationships and defining traits. It was noticed that the GSF and YSF genotypes were dispersed oppositely, indicating their significant genetic divergence for the traits under investigation. For PC-1, YSF genotypes exhibited high positive factor scores. Conversely, GSF genotypes exhibited negative factor scores. Furthermore, genotypes far from the origin demonstrated more variability for traits under study. It was observed that GSF genotypes were more inclined toward vectors of traits such as greenness index, color difference, chlorophyll content, 4-hydroxy isoleucine, free amino acid, days to 50% flowering, and harvest index as compared to remaining traits. Furthermore, it was interpreted that color contributing and biochemical traits such as 4-hydroxyisoleucine, chlorophyll, diosgenin, and total soluble sugar were major variability contributing traits (Figures 6B,C) (Supplementary Tables S3–S5).

### Trait correlation analysis

**Figure 7:**
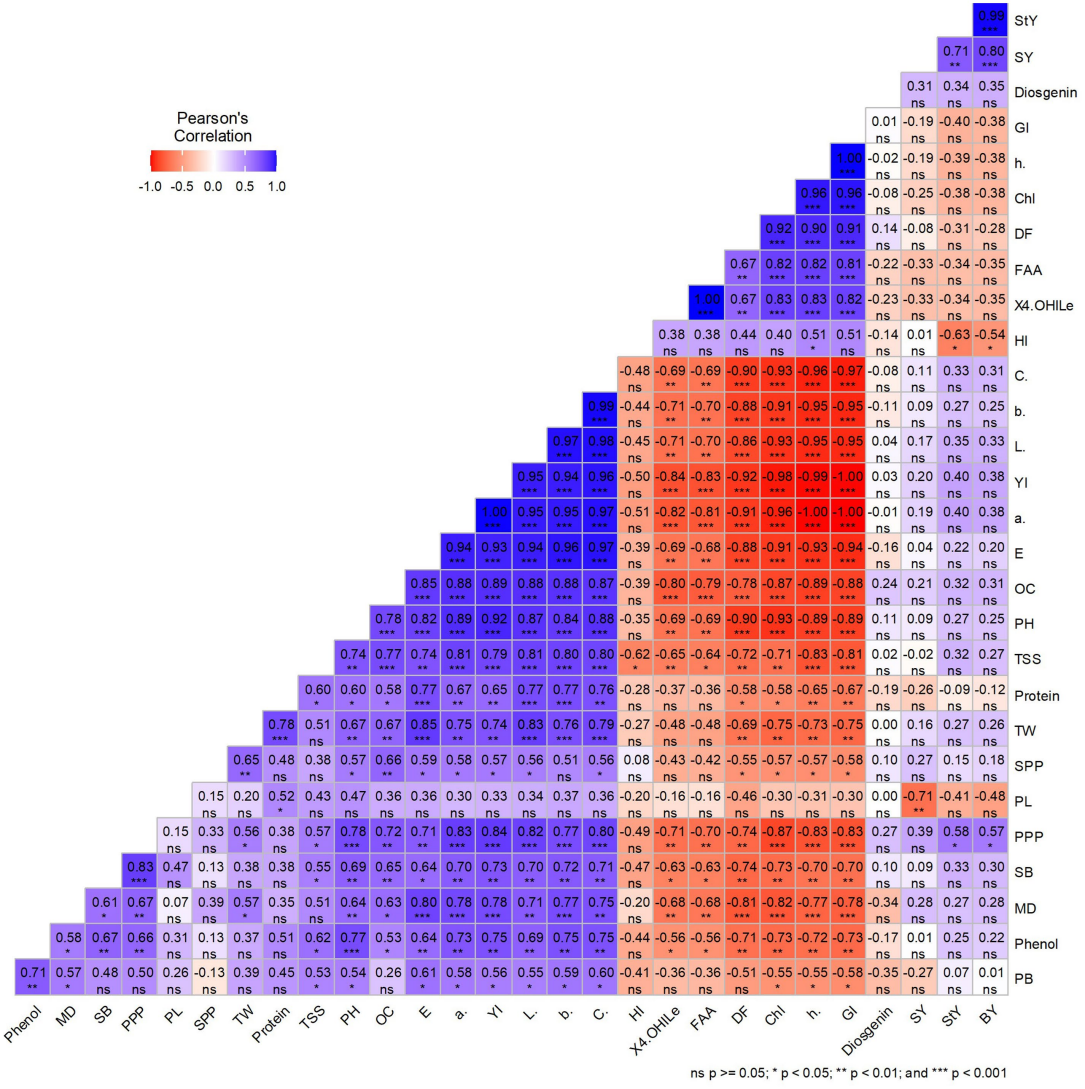
Correlation heat map for all studied traits among fenugreek genotypes PH (Plant height), PB (Primary branches per plant), SB (Secondary branches per plant), DF (Days to flowering), MD (Days to Maturity), PPP (Pods per plant), PL (Pod length), SPP (Seeds per pod), SY (Seed yield), BY (Biological yield), HI (Harvest index), TW (Test weight), 4-OHIle (4-Hydroxyisoleucine), TSS (Total soluble sugar), OC (Oil content), FAA (Free fatty acid), Chl (Chlorophyll content), L* (Lightness), a* (Red green axis), b* (Yellow blue axis), h (Hue), C (Chroma), E (Color difference), GI (Greeness index) YI (Yellowness index).

The worth of independent secondary traits in the selection process can be determined by their significant correlation with a dependent trait of interest. The correlation coefficient analysis was estimated to assess the relationship between the traits under investigation (Figure 7). 4-hydroxy isoleucine, chlorophyll content, free amino acid, hue, and greenness index were significantly positively correlated with each other. It appears that these traits are major contributors for enhancing the medicinal and nutritional status of fenugreek genotypes. An increase in chlorophyll content and greenness index is associated with higher level of 4-hydroxy isoleucine. Therefore, visually selecting fenugreek genotypes with green seeds may provide a promising option for addressing diabetes and obesity issues. Furthermore, it was observed that chroma, yellow blue axis, lightness, red green axis, color difference, oil content, plant height, total soluble sugar, pods per plant, secondary branches per plant, maturity days, and phenol content were significantly negatively correlated with 4-hydroxy isoleucine, free amino acid, days to flowering, chlorophyll content, hue, and greenness index. A significant negative correlation between 4-hydroxyisoleucine and total soluble sugar revealed their opposing behavior. This indicates that green-seeded fenugreek genotypes, with higher 4-OHIle will have lower TSS content, possess greater potential for human health benefits.

Yellow blue axis, chroma, lightness, yellowness index, red green axis, color difference, oil content, plant height, total soluble sugar, protein content, and test weight had significant positive correlation with each other. Number of seeds per pod depicted significant positive correlation with test weight, plant height, oil content, color difference, red green axis, yellowness index, lightness, and chroma, while it showed significant negative correlation with chlorophyll content, hue, and greenness index. Yellow-seeded fenugreek genotypes found to be agronomically better than green-seeded fenugreek genotypes; however, comparative performance difference for yield was not found to be so effective. The harvest index was higher in green-seeded genotypes due to differences in straw yield, highlighting their potential for commercial utilization too. In addition, there is scope to enhance the economic yield of green-seeded genotypes through the optimization of agronomical package of practices.

### Two-way clustering heat map and regression analysis

**Figure 8:**
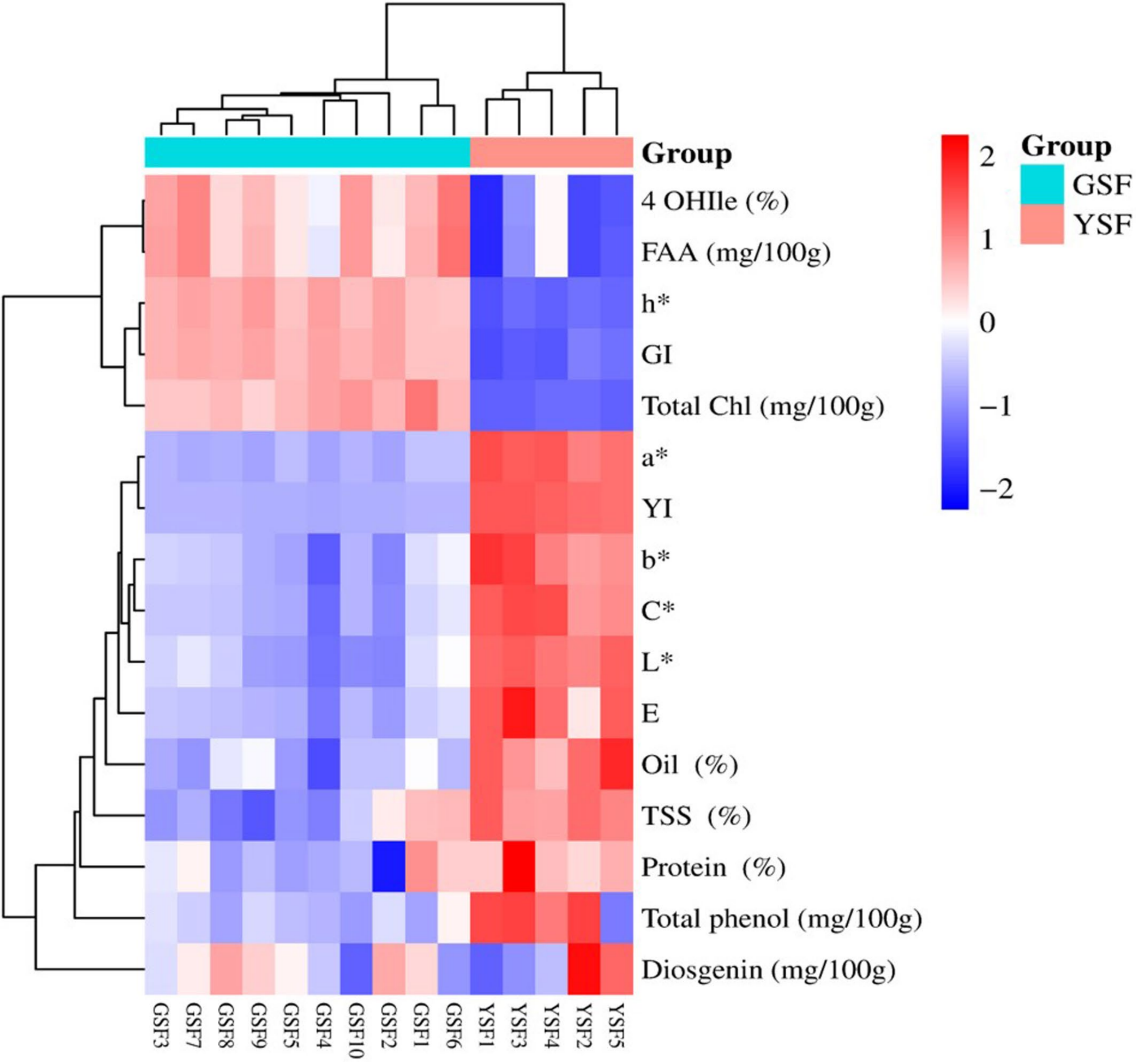
Two-way clustering heatmap for fenugreek genotype based on biochemical and seed color traits 4-OHIle (4-Hydroxyisoleucine), FAA (Free fatty acid), h* (Hue), GI (Greeness index), Total Chl (Total chlorophyll content), a*(Red green axis), YI (Yellowness index), b* (Yellow blue axis), C* (Chroma), L* (Lightness), E (Color difference), TSS (Total soluble sugar).

Data for biochemical and color traits along with experimental genotypes were subjected to two-way clustering heat map analysis, and a dendrogram was generated to visually inspect the clusters of genotypes. Two-way clustering heat map illustrating relationships between different traits (biochemical and color traits) (y-axis) regarding fenugreek genotypes estimated (x-axis) is shown in Figure 8. Colors within the heat map range from light blue (least prevalent) to dark red (most prevalent), illustrating the prevalence of a particular trait within a particular group. Clustering analysis was performed based upon the colors generated using complete cluster method and Euclidean distance. Cluster analysis cleaved 15 genotypes into two clear-cut distinct groups, i.e., GSF (green-seeded fenugreek) and YSF (yellow-seeded fenugreek) genotypes.

**Figure 9:**
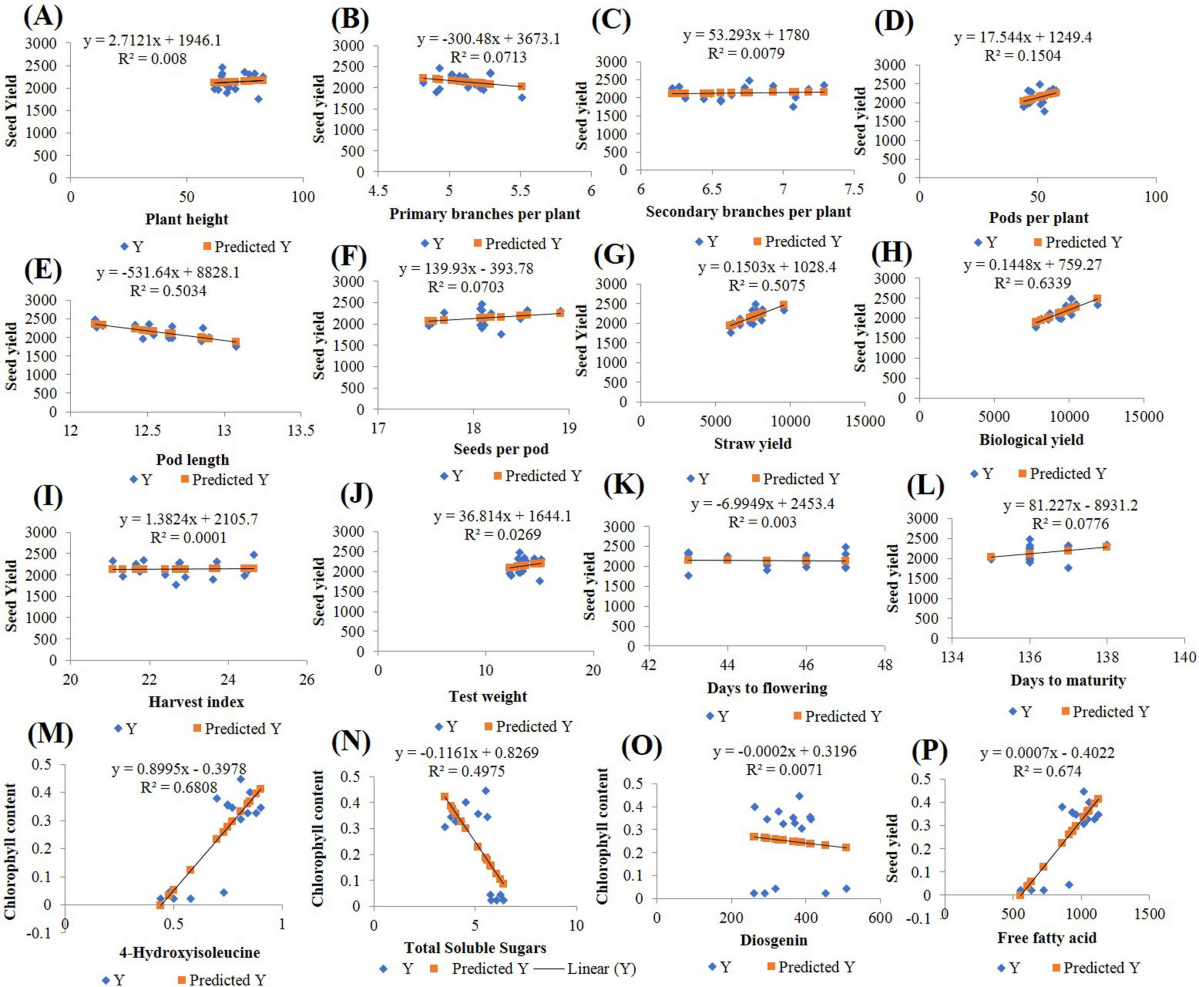
Regression analysis **(A)** Plant height **(B)** Primary branches per plant **(C)** Secondary branches per plant **(D)** Pods per plant **(E)** Pod length **(F)** Seeds per pod **(G)** Straw yield **(H)** Biological yield **(I)** Harvest index **(J)** Test weight **(K)** Days to flowering **(L)** Days to maturity **(M)** 4-Hydroxyisoleucine **(N)** Total soluble sugars **(O)** Disogenin **(P)** Free fatty acid.

The simple linear regression analysis was conducted to assess the relationship between yield-attributing traits (X-axis) and seed yield (Y-axis) and between biochemical traits (X-axis) and chlorophyll (Chl) content (Y-axis). The scatter plots, along with the fitted regression lines, are shown in Figure 9. The regression coefficients, with *p*-values <0.05 (alpha = 0.05), were found to be statistically significant for both the yield-contributing and biochemical traits. Among the agronomic traits, the biological yield (*R*^2^ = 63.39%) explained the greatest variation in seed yield. The graph indicates a positive linear relationship between biological yield and seed yield, with the regression line suggesting that as biological yield increases, seed yield also increases. The regression equation for this relationship is “Y = 759.27 + 0.1448X.” For the biochemical traits, 4-OHIle (*R*^2^ = 68.08%) and TSS (*R*^2^ = 49.75%) accounted for the highest proportions of variation in chlorophyll content. Both 4-OHIle and TSS showed linear relationships with chlorophyll content. Specifically, 4-OHIle exhibited a positive correlation, whereas TSS demonstrated a negative correlation with chlorophyll content. The regression equations for these relationships are “Y = −0.3978 + 0.8995X” for 4-OHIle and Y = 0.8269–0.1161X for TSS. These findings suggest that the biological yield and 4-OHIle significantly contribute to variations in seed yield and chlorophyll content, respectively.

## Discussion

Fenugreek is a potential source of medicinal compounds, but very limited variability is available in this crop. Hence, an experiment was conducted to develop a variety/genotype of the crop having higher concentrations of beneficial compounds. Fenugreek varieties, cultivars, and wild species are typically available with yellow color seeds, whereas green seeds are unusual. It has been proven that these green-seeded fenugreek genotypes show better performance in terms of medicinal properties as compared to their yellow counterparts (10) thereby making them an important natural dietary source for the individuals suffering from diabetes, obesity, insulin resistance, and tumor formation tendency in the body.

Obesity, diabetes, insulin resistance, and tumor formation are some of the common diseases affecting a large percentage of population. Few decades back, these diseases were thought to be correlated with aging but today we find small kids, teenagers, and youngsters also facing these issues. The major reason behind this may be an unhealthy lifestyle and unhealthy/poor diet. Other than regular diet, some natural supplements may offer additional nutrients necessary for the body. While synthetic medicines and supplements are available in the market as secure to the mentioned diseases, they come with their own share of ill side effects. Such is not the case with fenugreek, being a legume crop fenugreek is considered as an incredible source of protein, fiber, and minerals (4). The major component, which is very crucial to manage the insulin in body, is 4-hydroxyisoleucine (4-OHIle), and Fenugreek seeds are a rich source of 4-OHIle (44). Its consumption as medicine has been approved safe by United States Food and Drug Administration (FDA). This crop is rich not only in 4-OHIle but also in diosgenin, protein, free amino acids, total soluble sugar, and natural oil content (24).

To gain a deeper understanding of the agronomic and medicinal significance, an experiment was conducted in which both green- and yellow-seeded fenugreek genotypes were sown in the field during the rabi season across 3 consecutive years: 2021–22, 2022–23, and 2023–24. Sowing was done in 44th week for all the 3 years when the lowest temperature was 12 to 15°C and highest was 31 to 34°C with relative humidity (RH) 85–92% in morning. Germination took place within 4–5 days in all the genotypes. Data were recorded timely for all the agronomic traits, and significant differences were observed among the genotypes for all measured traits (*p < 0.01*, Table 2) suggesting variations among fenugreek genotypes. These results are consistent with the previous published reports by different researchers (45–47).

During different stages of crop growth, data were recorded for days to flowering (DF) and days to maturity (MD). After harvesting of fully matured crop, the data were recorded for PH, PB, SB, PPP, PL, SPP, SY, StY, BY, HI, and TW. Boxplot (Figure 3) analysis revealed consistent variation for the mentioned traits. The plant height of the fenugreek genotypes was significantly different among the cropping years which may be because of different weather conditions and rain patterns. Green-seeded fenugreek (GSF) genotypes showed statistical difference in comparison with yellow-seeded fenugreek (YSF) genotypes. The results are also consistent with the previous study by Sharma and Sastry (48) and Singh et al. (49) who reported average plant height in fenugreek genotypes as 49.8 cm and 65.91 cm, respectively. Similarly in the present experiment, average plant height ranges from 62 to 71 cm in GSF genotypes and 75 to 83 cm in YSF. In pooled data, the average plant height values were more in YSF genotypes as compared to GSF genotypes. When plants have increased number of vegetative parts and decreased row spacing, they achieve more height when compared to others. This situation can be explained by the increasing light competition (50). In addition, it has been stated that the height of plants decreases with the restriction of irrigation (51, 52). Similarly, in a previous research study, plant height was reported to be 24.95–85.15 cm (53) and 26.8 ± 14.9 cm in Omani fenugreek accessions (54). The findings showed that the number of primary branches is almost similar in most of the GSF and YSF genotypes barring a few. The branch number values were higher in 2022–23 than other growing years. This situation supports the opinion that precipitation and other environmental factors play important role in increasing the number of branches in fenugreek genotypes. These findings were partly similar to the results of Sharma and Sastry (48), who reported that branch numbers varied between 2.3 and 7.5 in 245 fenugreek genotypes. Similarly, the branch number of different fenugreek genotypes was found to be 2.18–7.98 (55), 2.40–4.90 (56), and 1.00–4.33 (57). Seeds per pod were comparable for both GSF and YSF genotypes over all three *rabi* seasons 2021–22, 2022–23, and 2023–24. Generally, it ranged from 17 to 19 in both fenugreek types. Singh et al. (58) reported number of pods/plant, pod length, and number of seeds/pod as 41.2, 9.47, and 16.67, respectively. In previous study by Desai et al. (59), highest number of pods/plant, pod length, and seeds/pod were reported to be 57.07, 12.57 cm, and 15.89 in *Pusa* Early Bunching. These results are consistent with our results. Similarly, Al-Maamari et al. (54) reported number of pods (32.1 ± 21.4), pod length (9.1 ± 1.2 cm), and number of seeds (134.2 ± 101.7) in fenugreek accessions. Camlica Mahmut and Gulsum (53) also observed the seeds/pod from 3.56 to 14.30 and pod length from 7.01 to 36.10 cm.

Maturity days were recorded, and it was reported that all fenugreek genotypes studied had a maturation duration time of 135–40 days. Pusa Early Bunching variety of fenugreek matured in 125–130 days. Singh et al. (60) claimed an extra early maturing accession *IC 0624520* that matured in 120 to 140 days. It was observed in our study that green-seeded genotypes took 3–4 more days in 50% flowering when compared to YSF. Bhutia et al. (61) reported that flowering took place in approximately 49.25 days in fenugreek, whereas Sultana et al. (62) reported that 50% flowering took place in 44.60 days.

Seed yield showed significant differences in the *rabi* periods of 2021–22, 2022–23, and 2023–24 among the fenugreek genotypes grown under normal condition. Some of the main reasons for the difference in seed yield between years and genotypes is the difference in environmental and genetic factors during the vegetation period (63). It was reported that morphological properties such as plant height and branch number can be closely related to seed yield in fenugreek (64). It has been reported that the seed yield is higher in fenugreek genotypes with higher plant height, maturation time, and biological yield (65), but our GSF genotypes showed better seed yield in comparison with YSF genotypes although the plant height of GSF genotypes is less than the YSF genotypes. This may be because of the bold size of the seeds of GSF. In addition, it was also reported that seed yield varies depending on years, sowing date, harvest date, climatic conditions, and irrigation (66). In a study for standardization of organic module for sustainable production of fenugreek, the observed number of branches, number of pods, number of seeds, and highest grain yield were 6.76 per plant, 42 pods/plant, 16.01 seed/pod, and 1515.21 kg/ha, respectively (67).

Straw yield reported to be higher for YSF genotypes as compared to GSF genotypes indicating more photosynthates accumulation. Singh et al. (58) reported straw yield (4,954 kg/ha), which is almost half of our reported straw yield. This may be because of environment, genotype, and date of sowing variability. Biological yield of fenugreek genotypes can vary significantly, depending on the genotypes and the environmental conditions. Seed yield, biological yield, and harvest index of the green-seeded fenugreek genotypes are more in comparison with the yellow-seeded fenugreek genotypes, favoring the GSF genotypes in terms of the agronomic traits. The data showed that GSF genotypes performed better over YSF for PL, SY, StYBY, HI, and TW when lesser rains (624.7 mm in 2021–22) were received in comparison with 2022 to 2024 (915.3 mm and 996.0 mm). Further studies can reveal its adaptability to moisture stress. Given the medicinal potential of the green-seeded genotypes compared to the yellow-seeded ones, their slight yield loss is not a major concern. This can be offset by the higher commercial value of the GSF genotypes, making them a favorable choice for farmers in monetary terms while providing greater benefits from the same resources.

Yield is one of the important characters for any genotype/variety, but spices and medicinal crops are better judged based on the medicinal potential rather than the yield. In our experiment, all the tested GSF genotypes are superior to YSF genotypes in medicinal properties. The most abundant free amino acid in fenugreek, 4-hydroxyisoleucine, belongs to the category of amino acid derivatives or bioactive compounds but not secondary metabolites. It is primarily known for its role in modulating insulin secretion and influencing glucose metabolism and acts as a precursor for insulin secretion, which has made it a subject of study in diabetes management. This amino acid is primarily found in the seed endosperm of fenugreek (43), and its abundance in the seeds contributes to the nutritional and medicinal value of the plant. Previous literature reports a wide range of 4-OHIle concentrations in fenugreek seeds, ranging from 0.015 to 0.4% (44, 68, 69). Haeri et al. (70) reported that germinated fenugreek seeds contain roughly double 4-OHIle as compared to dry fenugreek seeds. Rajabihashjin et al. (71) had suggested that temperature and solar irradiation play significant roles in the accumulation of 4-OHIle. 4-OHIle may be a potential treatment for insulin resistance by regulating blood glucose, lipotoxic reducer, liver function enhancer, and obesity inhibitor. GSF6, GSF7, GSF10, and GSF3 can be rich source for antidiabetic and anti-obesity effects having almost 1.5 to double amount of 4-OHIle in comparison with YSF and other reported genotypes by different researchers. 4-OHIle exerts its effect by enhancing Akt phosphorylation and reducing the stimulation of Jun N-terminal kinase (JNK) 1/2, extracellular signal regulated kinase (ERK) 1/2, p38 mitogen activated protein kinase (MAPK), and nuclear factor (NF)-κB (24).

Chlorophyll-rich food is associated with brain health, anti-cancer properties, neuroprotective, endocrine disruptor effects, and anti-obesity effects with ample of antioxidant properties (26). Its unique structure assists it to scavenge harmful free radicles, DNA damage repairment, and cellular process regulation (72–74). In a study, it was found that *in vivo* absorption of chlorophyll derivative using SCC tablets (300 mg/day) resulted in absorption of Cu-chlorin e4 ethyl ester in human gastrointestinal tract (75). Matured seeds of fenugreek contain the raffinose family oligosaccharides (RFOs) as the major storage sugars, i.e., 65.37 mg g^−1^ DW (76). Total soluble sugar plays a negative role for the diabetic persons. GSF genotypes are reported to be lower in TSS in comparison with YSF genotypes, favoring the consumption of GSF for sugar patients or health-conscious persons. The amount and type of carbohydrates in the diet impact blood glucose levels, which in turn affect insulin release and the rate of gastric emptying (77).

Diosgenin (*25R-spirost-en-3β-ol*), a steroidal compound, plays an important role in diabetes cure by cellular pathway modulation. It works by reducing intestinal glucose uptake, decreasing metabolism in organ and tissues, increasing insulin secretion, and improving insulin resistance. Diabetes alters pathways such as *serine/threonine protein kinase* and *protein kinase C /glucose transporter 4*, and peroxisome proliferator-activated receptor glucose absorption, and inhibits *α-amylase* and *α-glucosidase*, *sodium/glucose cotransporter 1,* and *Na^+^ K^+^ ATPase* activity. Diabetes may also be caused by a decrease in the expression of *sterol regulatory element binding protein 1* and its target genes, *fatty acid synthase*, *stearoyl-CoA desaturase-1,* and *acetyl-CoA carboxylase α* as well as a decrease in the levels of *C/EBP homologous protein*, *Caspase12,* and *Caspase3* proteins (78). Moreover, diosgenin possesses anticarcinogenic properties which reduce tumor cell proliferation and triggering apoptosis (79). But as studies by Khosravi et al. (80) show, overdose of diosgenin may cause male infertility. Diosgenin ranges from 0.1 to 0.90% in seeds (81). Diosgenin content is low in GSF10, supporting its consumption for males who are consuming fenugreek seeds for other health benefits.

FAAs, water-soluble compounds, are key quality signs of processing and storage conditions. In addition, some free amino acids contribute to taste and aroma by participating in Maillard reactions through the generation of volatile compounds (82). Higher amount of free amino acids in GSF is an additional feature of green-seeded fenugreek genotypes further enhancing its benefits.

Total phenol has antioxidant properties, i.e., preventive in chronic illnesses such as cancer, diabetes, cardiovascular disease, and neurodegenerative diseases (83, 84). Reactive oxygen species (ROS) includes superoxide, peroxide, singlet oxygen, nitric oxide, hydroxyl radical, and peroxynitrite that are produced during impaired antioxidant system leading to cellular damage (85). Researchers have indicated that fenugreek contains 6–8% (86), 6.7% (87), 8.94% (88), 3.25–6.88% (89), and 8% (90) oil content. Our study showed that the oil content is almost in similar range (3.0 to 4.5%) in both the GSF and YSF genotypes. Many researchers have reported fenugreek seed protein content ranging from 20–26% (86, 91, 92). In our study, YSF genotypes showed protein levels of 21–25%, higher than the 15.7–21.7% found in GSF genotypes.

Seed color is vital in breeding due to its role in germination, dormancy, and stress resistance (93, 94), serving as marker for genetic purity and seed quality (95). It significantly also impacts or influences marketability as uniform and desired appealing color meet consumer preferences and fetch premium prices. Integrating seed color traits ensures better performance, quality, and acceptance. The current seed chromatic analysis distinctly categorized different fenugreek genotypes into two distinct groups, i.e., yellow- and green-seeded. The GSF1 has greenest/darkest green color seeds with highest amount of chlorophyll among studied genotypes. YSF1 and YSF3 had maximum yellow pigment. Based on the values for L*a*b*, seed color code and color shade name were identified which revealed that the green-seeded fenugreek genotypes had three different shades of green color (dark olive green, dark slate gray, and dim gray), whereas all the five YSF genotypes had saddle brown shade of yellow color (Table 3). The findings showed wider diversity in terms of degree of greenness and yellowness among the GSF and YSF genotypes. Hence, these genotypes could be used as donor parents for incorporating and introgression of green, yellow, or intermediate color types of fenugreek seeds. In addition, seed color also symbolizes quality, and traits such as nutritional content as pigments like carotenoids and tannins contributed to both color and health benefits (96). The information generated in this study could also be utilized for identifying genotypes having higher levels of medicinal compounds, antioxidants, and phenolic and essential nutrients offering enhanced protection against oxidative stress. Understanding relationship between seed color and nutrition further allows breeders to develop varieties that combine aesthetic appeal with improved nutritional profiles meeting out both consumer demand and health objectives.

## Conclusion

In the present study, significant variability was observed for agro-morphological traits, biochemical components, and color content in both types of the fenugreek genotypes. The findings showed that the green-seeded genotypes are the potential source for medicinal values with good amount of biological yield and harvest index. The yield of green-seeded fenugreek genotypes can be improved with more rigorous agronomic practices. GSF genotypes are rich source of 4-hydroxyisoleucine (4-OHIle%), which may play a very important role in insulin resistance and obesity. The green fenugreek seeds contain 1.5 to double amount of 4-OHIle and can be potential natural source as medicine for diabetes and obesity. Genotype GSF6 (IC-0633367) is rich in 4-OHIle (0.90%), and genotype GSF9 (IC-0633370) has lowest amount of TSS with good amount of 4-OHIle (0.81%). Genotype GSF1 (IC-0633362) is rich in Chl content with darkest green color, good amount of 4-OHIle, oil, and protein. Genotype GSF8 (IC-0633369) showed better seed yield (2473.74 kg ha^1^) and harvest index (24.65%). Green-seeded genotypes have been reported to be particularly valuable in future breeding projects for better fenugreek varieties, as well as in the medical, pharmaceutical, and ayurvedic industries. It is essential to study the agronomic and yield characteristics of fenugreek genotypes under various agro-ecological conditions to fully understand and further strengthen their potential and variability. In addition, exploring the genetic basis of these traits could uncover novel genes and pathways that can be targeted to enhance future breeding efforts and optimize the crop’s therapeutic and agricultural value.

## Data Availability

The original contributions presented in the study are included in the article/Supplementary material, further inquiries can be directed to the corresponding authors.
