# Supplementary material for: Deciphering agronomic traits, biochemical components, and color in unique green-seeded fenugreek (Trigonella foenum-graecum L.) genotypes
Source: Front Nutr. 2025 Feb 27;12:1542211. doi: 10.3389/fnut.2025.1542211 (PMC11903288; doi:10.3389/fnut.2025.1542211)
Supplement: Supplementary file 1 [file Table_1.docx]

**TABLE S1 Details of 10 green seeded fenugreek genotypes used**

| **Genotype/ Variety** | **Genotypes** | **Seed Color** | **Collector No.** | **Site of collection** | **Year of collection** |
| --- | --- | --- | --- | --- | --- |
| IC-0633362 | GSF1 | Green | RS/SC/FGS/14-1 | Khajwa, Nagaur, Rajasthan | 2014 |
| IC-0633363 | GSF2 | Green | RS/SC/FGS/14-2 | Mundawa, Nagaur, Rajasthan | 2014 |
| IC-0633364 | GSF3 | Green | RS/SC/FGS/14-3 | Khen, Nagaur, Rajasthan | 2014 |
| IC-0633365 | GSF4 | Green | RS/SC/FGS/14-4 | Mundawa, Nagaur, Rajasthan | 2014 |
| IC-0633366 | GSF5 | Green | RS/SC/FGS/14-5 | Inana, Nagaur, Rajasthan | 2014 |
| IC-0633367 | GSF6 | Green | RS/SC/FGS/14-6 | Mundawa, Nagaur, Rajasthan | 2014 |
| IC-0633368 | GSF7 | Green | RS/SC/FGS/15-7 | Dhainda, Nagaur, Rajasthan | 2015 |
| IC-0633369 | GSF8 | Green | RS/SC/FGS/15-8 | Janana, Nagaur, Rajasthan | 2015 |
| IC-0633370 | GSF9 | Green | RS/SC/FGS/15-9 | Kharda, Nagaur, Rajasthan | 2015 |
| IC-0633371 | GSF10 | Green | RS/SC/FGS/15-10 | Bhadana, Nagaur, Rajasthan | 2015 |

**TABLE S2 Meteorological data for 2021-22, 2022-23 and 2023-24**

|  | **Standard week No (2021-22)** | **Temperature** | | **Relative humidity (%)** | | **Evapo mm/day** | **Total weekly rainfall (mm)** |
| --- | --- | --- | --- | --- | --- | --- | --- |
|  |  | **Minimum** | **Maximum** | **7.40 am** | **2.40pm** |  |  |
| **2021 Oct to Dec** | 40 | 24.3 | 33.1 | 90.7 | 75.6 | 2.4 | 24.6 |
|  | 41 | 24.8 | 35.4 | 85.6 | 57.7 | 7.4 |  |
|  | 42 | 17.6 | 34.3 | 89.0 | 50.6 | 6.1 |  |
|  | 43 | 16.9 | 32.4 | 90.9 | 57.1 | 4.1 |  |
|  | 44 | 12.3 | 31.0 | 85.9 | 57.6 | 5.1 |  |
|  | 45 | 10.0 | 31.4 | 88.3 | 51.9 | 5.4 |  |
|  | 46 | 9.1 | 30.6 | 86.3 | 56.1 | 5.9 |  |
|  | 47 | 11.3 | 25.3 | 87.0 | 72.7 | 3.5 | 3.0 |
|  | 48 | 10.1 | 30.8 | 87.9 | 47.9 | 4.0 |  |
|  | 49 | 10.7 | 25.3 | 88.1 | 70.3 | 2.1 |  |
|  | 50 | 6.9 | 25.4 | 89.1 | 66.0 | 2.5 |  |
|  | 51 | 5.0 | 22.1 | 88.9 | 62.6 | 2.1 |  |
|  | 52 | 7.4 | 25.4 | 90.3 | 44.1 | 2.4 |  |
| **2022 Jan to April** | 1 | 7.6 | 20.6 | 89.4 | 61.4 | 1.4 |  |
|  | 2 | 11.0 | 19.0 | 90.7 | 71.6 | 1.4 | 15 |
|  | 3 | 3.4 | 18.6 | 90.0 | 56.4 | 2.2 |  |
|  | 4 | 7.0 | 21.9 | 88.6 | 56.6 | 2.1 | 6 |
|  | 5 | 2.1 | 22.7 | 91.1 | 52.0 | 2.4 |  |
|  | 6 | 8.3 | 25.6 | 87.0 | 46.7 | 3.6 |  |
|  | 7 | 6.7 | 25.9 | 85.9 | 47.4 | 3.6 |  |
|  | 8 | 7.1 | 29.6 | 86.3 | 48.7 | 5.4 |  |
|  | 9 | 11.1 | 30.7 | 87.3 | 51.1 | 6.9 |  |
|  | 10 | 9.1 | 29.9 | 88.0 | 50.3 | 6.1 |  |
|  | 11 | 13.4 | 32.9 | 89.4 | 36.8 | 8.1 |  |
|  | 12 | 18.6 | 37.3 | 73.6 | 35.3 | 10.5 |  |
|  | 13 | 17.2 | 36.6 | 81.9 | 36.5 | 9.4 |  |
|  | 14 | 19.3 | 39.3 | 82.7 | 35.9 | 11.3 |  |
|  | 15 | 19.0 | 41.2 | 82.3 | 31.5 | 13.6 |  |
|  | 16 | 23.1 | 40.0 | 82.4 | 57.7 | 12.3 |  |
|  | 17 | 23.6 | 40.2 | 86.7 | 63.7 | 12.9 |  |
| **2022 Oct to Dec** | 40 | 24.1 | 34.9 | 91.0 | 59.7 | 6.9 |  |
|  | 41 | 23.0 | 32.2 | 93.0 | 73.3 | 4.4 | 10.0 |
|  | 42 | 18.7 | 33.5 | 94.4 | 55.4 | 5.2 |  |
|  | 43 | 15.0 | 33.9 | 91.1 | 41.4 | 7.0 |  |
|  | 44 | 15.4 | 33.5 | 90.7 | 47.1 | 7.7 |  |
|  | 45 | 14.1 | 34.6 | 90.1 | 43.6 | 6.3 |  |
|  | 46 | 14.4 | 30.9 | 89.4 | 54.7 | 5.3 |  |
|  | 47 | 10.6 | 28.9 | 89.7 | 58.3 | 5.4 |  |
|  | 48 | 7.0 | 28.6 | 89.4 | 53.4 | 5.5 |  |
|  | 49 | 7.6 | 26.4 | 88.7 | 65.7 | 5.1 |  |
|  | 50 | 6.9 | 25.9 | 87.3 | 66.4 | 5.1 |  |
|  | 51 | 7.9 | 27.4 | 87.7 | 62.6 | 5.3 |  |
|  | 52 | 7.3 | 24.5 | 88.4 | 77.3 | 3.6 |  |
|  | 53 | 4.7 | 24.3 | 87.6 | 53.0 | 3.4 |  |
| **2023 Jan to April** | 1 | 2.7 | 21.3 | 86.7 | 72.6 | 2.9 |  |
|  | 2 | 6.6 | 24.1 | 86.3 | 61.6 | 3.9 |  |
|  | 3 | 4.9 | 20.4 | 87.7 | 55.1 | 2.8 |  |
|  | 4 | 5.4 | 21.0 | 88.9 | 52.3 | 3.0 | 28 |
|  | 5 | 6.3 | 23.9 | 86.7 | 60.6 | 3.0 | 24 |
|  | 6 | 10.1 | 26.7 | 90.3 | 69.6 | 5.6 |  |
|  | 7 | 7.7 | 31.4 | 86.7 | 58.8 | 6.8 |  |
|  | 8 | 13.3 | 31.6 | 88.4 | 43.6 | 8.1 |  |
|  | 9 | 14.7 | 32.1 | 88.9 | 52.1 | 7.8 | 8.0 |
|  | 10 | 14.1 | 31.7 | 88.6 | 47.1 | 7.1 |  |
|  | 11 | 16.3 | 32.1 | 89.4 | 52.4 | 6.9 |  |
|  | 12 | 16.3 | 30.0 | 89.4 | 66.3 | 6.4 |  |
|  | 13 | 17.4 | 30.4 | 89.9 | 63.3 | 8.2 | 15.0 |
|  | 14 | 20.9 | 33.6 | 90.7 | 62.1 | 11.8 |  |
|  | 15 | 25.4 | 38.0 | 70.9 | 52.4 | 18.8 |  |
|  | 16 | 25.7 | 35.9 | 66.3 | 58.9 | 20.0 |  |
|  | 17 | 22.9 | 33.7 | 84.1 | 61.4 | 21.1 |  |
| **2023 Oct to Dec** | 40 | 20.9 | 35.4 | 91.0 | 46.4 | 9.1 |  |
|  | 41 | 22.1 | 34.9 | 82.9 | 50.9 | 8.4 |  |
|  | 42 | 17.9 | 33.1 | 87.1 | 48.0 | 7.6 |  |
|  | 43 | 16.6 | 33.7 | 88.1 | 41.7 | 7.5 |  |
|  | 44 | 14.7 | 33.5 | 88.9 | 43.9 | 8.5 |  |
|  | 45 | 13.7 | 33.3 | 88.4 | 42.6 | 8.4 |  |
|  | 46 | 10.9 | 33.0 | 87.1 | 38.4 | 8.3 |  |
|  | 47 | 9.4 | 28.1 | 87.6 | 62.6 | 6.1 |  |
|  | 48 | 9.9 | 23.9 | 85.9 | 71.9 | 4.0 | 3.0 |
|  | 49 | 5.7 | 19.1 | 85.7 | 68.6 | 2.8 |  |
|  | 50 | 4.9 | 18.1 | 87.3 | 73.7 | 2.0 |  |
|  | 51 | 6.4 | 19.3 | 87.3 | 66.1 | 2.0 |  |
|  | 52 | 6.9 | 20.9 | 83.4 | 56.6 | 3.5 |  |
| **2024 Jan to April** | 1 | 5.7 | 20.1 | 84.4 | 66.9 | 3.6 |  |
|  | 2 | 4.7 | 20.4 | 83.7 | 69.9 | 2.4 |  |
|  | 3 | 8.3 | 20.0 | 85.9 | 70.7 | 2.1 |  |
|  | 4 | 6.4 | 24.7 | 84.7 | 55.0 | 3.2 |  |
|  | 5 | 8.6 | 24.6 | 85.9 | 52.1 | 4.8 |  |
|  | 6 | 7.4 | 23.6 | 85.1 | 55.6 | 2.7 | 18 |
|  | 7 | 8.7 | 23.6 | 87.3 | 65.3 | 5.4 |  |
|  | 8 | 11.0 | 24.4 | 84.4 | 69.4 | 8.1 |  |
|  | 9 | 12.9 | 23.9 | 75.3 | 70.6 | 6.1 |  |
|  | 10 | 9.3 | 26.9 | 86.4 | 62.9 | 8.9 |  |
|  | 11 | 14.7 | 31.3 | 80.3 | 54.0 | 10.0 |  |
|  | 12 | 18.7 | 35.3 | 63.0 | 37.1 | 11.4 |  |
|  | 13 | 21.9 | 37.1 | 43.1 | 25.1 | 12.5 |  |
|  | 14 | 25.4 | 35.9 | 54.3 | 42.7 | 11.4 |  |
|  | 15 | 27.1 | 34.1 | 49.0 | 40.3 | 13.9 | 8.0 |
|  | 16 | 27.0 | 37.8 | 42.0 | 39.1 | 13.8 |  |
|  | 17 | 27.3 | 39.1 | 44.9 | 40.6 | 15.7 |  |
|  | 18 | 26.3 | 38.6 | 38.9 | 45.9 | 16.4 |  |

**TABLE S3 Eigen value, percentage of variance, cumulative percentage of variance of principal components and percentage contribution of all variables on PCs**

| **SN** | **Variables** | **PC1** | **PC2** | **PC3** |
| --- | --- | --- | --- | --- |
| **1.** | **Eigen value** | 17.409 | 3.667 | 1.922 |
| **2.** | **Percentage of variance** | 62.175 | 13.096 | 6.864 |
| **3.** | **Cumulative percentage of variance** | 62.175 | 75.271 | 82.135 |
| **SN** | **Variables** | **PC1** | **PC2** | **PC3** |
| **1** | **Plant height (PH)** | 4.366 | 0.257 | 0.318 |
| **2** | **Primary branches per plant (PB)** | 1.734 | 2.627 | 16.794 |
| **3** | **Secondary branches per plant (SB)** | 2.972 | 0.004 | 2.296 |
| **4** | **Days to 50% Flowering (DF)** | 4.439 | 0.315 | 0.009 |
| **5** | **Maturity days (MD)** | 3.187 | 0.023 | 0.161 |
| **6** | **Pods per plant (PPP)** | 3.788 | 2.42 | 0.429 |
| **7** | **Pod length (PL)** | 0.646 | 13.819 | 0.021 |
| **8** | **Seeds per pod (SPP)** | 1.622 | 0.025 | 21.49 |
| **9** | **Seed yield (gm/ha)** | 1.55 | 0.729 | 12.594 |
| **10** | **Seed yield (Kg/ha)** | 0.172 | 21.129 | 2.062 |
| **11** | **Straw yield (StY)** | 0.797 | 18.895 | 3.731 |
| **12** | **Biological yield (BY)** | 0.715 | 21.014 | 1.973 |
| **13** | **Harvest index (HI)** | 1.325 | 1.405 | 15.807 |
| **14** | **Test weight (TW)** | 3.05 | 0.089 | 1.697 |
| **15** | **4-hydroxy isoleucine (4-OHILe)** | 3.445 | 1.053 | 1.58 |
| **16** | **Total soluble sugar (TSS)** | 3.406 | 0.171 | 2.078 |
| **17** | **Oil content (OC)** | 4.178 | 0.083 | 3.326 |
| **18** | **Protein** | 2.422 | 6.129 | 0.131 |
| **19** | **Diosgenin** | 0.004 | 6.473 | 5.754 |
| **20** | **Free fatty acid (FAA)** | 3.402 | 1.045 | 1.43 |
| **21** | **Phenol** | 2.965 | 0.449 | 5.65 |
| **22** | **Total chlorophyll content (Chl)** | 4.986 | 0.069 | 0.384 |
| **23** | **Lightness (L*)** | 4.892 | 0.041 | 0.003 |
| **24** | **Red Green Axis (a*)** | 5.149 | 0.004 | 0.001 |
| **25** | **Yellow Blue Axis (b*)** | 4.86 | 0.479 | 0.106 |
| **26** | **Hue (h*)** | 5.103 | 0.013 | 0.02 |
| **27** | **Chroma (C*)** | 4.994 | 0.218 | 0.081 |
| **28** | **Color Difference (ΔE*)** | 4.653 | 0.865 | 0.015 |
| **29** | **Greeness index (GI)** | 5.149 | 0.004 | 0.001 |
| **30** | **Yellowness index (YI)** | 5.184 | 0.012 | 0.039 |

**TABLE S4 Factor loadings of Variables**

| SN | **Variables** | **PC1** | **PC2** | **PC3** | **PC4** | **PC5** | **PC6** | **PC7** | **PC8** | **PC9** | **PC10** | **PC11** | **PC12** | **PC13** | **PC14** | **PC15** |
| --- | --- | --- | --- | --- | --- | --- | --- | --- | --- | --- | --- | --- | --- | --- | --- | --- |
| **1** | **PH** | 0.209 | 0.051 | 0.056 | -0.091 | 0.011 | -0.216 | -0.02 | -0.186 | -0.293 | -0.299 | 0.229 | -0.08 | 0.166 | -0.246 | -0.281 |
| **2** | **PB** | 0.132 | 0.162 | -0.41 | 0.058 | 0.19 | -0.022 | 0.398 | 0.036 | -0.035 | -0.436 | -0.013 | 0.224 | -0.109 | -0.026 | 0.129 |
| **3** | **SB** | 0.172 | -0.007 | -0.152 | -0.274 | 0.119 | -0.298 | -0.269 | 0.389 | 0.106 | 0.281 | 0.183 | 0.274 | -0.148 | -0.344 | -0.17 |
| **4** | **DF** | -0.211 | -0.056 | 0.009 | -0.024 | -0.061 | 0.128 | 0.377 | 0.131 | -0.101 | 0.137 | -0.19 | 0.261 | -0.168 | 0.006 | -0.347 |
| **5** | **MD** | 0.179 | -0.015 | -0.04 | 0.223 | 0.387 | 0.055 | -0.229 | 0.21 | 0.173 | -0.189 | -0.034 | -0.163 | -0.059 | 0.458 | -0.585 |
| **6** | **PPP** | 0.195 | -0.156 | -0.065 | -0.131 | 0.078 | -0.34 | 0.08 | 0.17 | 0.058 | -0.017 | -0.262 | 0.324 | 0.229 | 0.378 | 0.254 |
| **7** | **PL** | 0.08 | 0.372 | 0.014 | -0.361 | -0.203 | -0.122 | -0.322 | 0.065 | 0.059 | -0.087 | 0.235 | 0.008 | 0.025 | 0.211 | 0.099 |
| **8** | **SPP** | 0.127 | -0.016 | 0.464 | 0.223 | -0.183 | 0.01 | -0.239 | -0.222 | -0.118 | -0.169 | 0.031 | 0.478 | -0.342 | 0.161 | -0.003 |
| **9** | **SY (gm/ha)** | 0.124 | 0.085 | 0.355 | -0.274 | 0.28 | -0.126 | 0.138 | -0.465 | 0.265 | 0.127 | -0.207 | -0.064 | 0.175 | -0.004 | -0.123 |
| **10** | **SY (Kg/ha)** | 0.042 | -0.46 | 0.144 | 0.185 | 0.143 | -0.079 | 0.013 | 0.096 | -0.184 | 0.215 | 0.208 | -0.131 | 0.271 | -0.03 | 0.005 |
| **11** | **BY** | 0.085 | -0.458 | -0.14 | 0.061 | -0.111 | -0.083 | -0.088 | -0.133 | 0.036 | -0.004 | 0.083 | 0.065 | -0.065 | 0.038 | 0.045 |
| **12** | **HI** | -0.115 | 0.119 | 0.398 | 0.211 | 0.311 | -0.227 | 0.058 | 0.294 | -0.402 | -0.073 | 0.071 | 0.127 | 0.02 | -0.08 | 0.151 |
| **13** | **TW** | 0.175 | 0.03 | 0.13 | 0.297 | -0.234 | -0.146 | 0.262 | 0.096 | 0.345 | -0.152 | 0.371 | -0.264 | -0.083 | -0.136 | 0.036 |
| **14** | **4-OHILe** | -0.186 | 0.103 | -0.126 | 0.2 | -0.209 | -0.391 | -0.134 | -0.096 | -0.067 | -0.056 | -0.248 | -0.077 | 0.066 | -0.037 | -0.142 |
| **15** | **TSS** | 0.185 | 0.041 | -0.144 | -0.061 | -0.234 | 0.404 | -0.137 | 0.05 | -0.409 | -0.12 | 0.044 | 0.013 | 0.308 | 0.095 | -0.091 |
| **16** | **OC** | 0.204 | -0.029 | 0.182 | -0.079 | -0.118 | 0.168 | -0.132 | 0.196 | -0.097 | 0.096 | -0.344 | -0.281 | -0.309 | -0.019 | 0.147 |
| **17** | **Protein** | 0.156 | 0.248 | 0.036 | 0.163 | -0.294 | -0.03 | 0.297 | -0.02 | 0.069 | 0.435 | 0.251 | 0.199 | 0.11 | 0.309 | -0.151 |
| **18** | **Diosgenin** | 0.006 | -0.254 | 0.24 | -0.48 | -0.299 | -0.149 | 0.325 | 0.208 | -0.098 | -0.259 | -0.065 | -0.147 | -0.133 | 0.081 | -0.24 |
| **19** | **FAA** | -0.184 | 0.102 | -0.12 | 0.199 | -0.221 | -0.393 | -0.135 | -0.088 | -0.076 | -0.043 | -0.286 | -0.092 | 0.064 | 0.003 | -0.092 |
| **20** | **Phenol** | 0.172 | 0.067 | -0.238 | -0.091 | 0.201 | -0.171 | 0.123 | -0.341 | -0.443 | 0.276 | 0.148 | -0.19 | -0.443 | 0.165 | -0.016 |
| **21** | **Chl** | -0.223 | 0.026 | -0.062 | 0.014 | -0.098 | 0.092 | -0.043 | 0.019 | -0.03 | 0.163 | 0.001 | 0.178 | 0.013 | -0.191 | -0.323 |
| **22** | **L*** | 0.221 | 0.02 | 0.006 | 0.076 | -0.121 | -0.062 | 0.095 | 0.157 | -0.093 | 0.074 | -0.039 | -0.106 | 0.199 | 0.124 | 0.068 |
| **23** | **a*** | 0.227 | -0.006 | 0.003 | 0.03 | 0.016 | 0.076 | 0.006 | -0.082 | 0.019 | -0.014 | -0.101 | 0.14 | 0.075 | -0.143 | -0.037 |
| **24** | **b*** | 0.22 | 0.069 | -0.033 | 0.09 | -0.028 | 0.005 | 0.001 | 0.124 | -0.078 | 0.217 | -0.18 | -0.162 | -0.041 | 0.026 | 0.125 |
| **25** | **h** | -0.226 | 0.011 | -0.014 | -0.004 | -0.004 | -0.108 | 0 | 0.073 | 0.005 | 0.016 | 0.149 | -0.117 | -0.214 | 0.201 | 0.058 |
| **26** | **C** | 0.223 | 0.047 | -0.028 | 0.097 | -0.06 | -0.056 | 0.002 | 0.004 | -0.041 | 0.094 | -0.198 | -0.026 | -0.042 | -0.151 | -0.009 |
| **27** | **ΔE*** | 0.216 | 0.093 | 0.012 | 0.165 | -0.025 | 0.043 | 0.014 | 0.113 | 0.157 | -0.056 | -0.139 | -0.013 | -0.265 | -0.192 | 0.001 |
| **28** | **GI** | -0.227 | 0.006 | -0.003 | -0.03 | -0.016 | -0.076 | -0.006 | 0.082 | -0.019 | 0.014 | 0.101 | -0.14 | -0.075 | 0.143 | 0.037 |
| **29** | **E** | -0.22 | -0.037 | 0.015 | -0.103 | 0.136 | 0.117 | -0.068 | -0.054 | 0.079 | -0.056 | 0.16 | 0.039 | -0.086 | 0.096 | 0.161 |
| **30** | **YI** | 0.228 | -0.011 | 0.02 | -0.012 | 0.029 | 0.035 | 0.002 | -0.084 | 0.017 | -0.018 | 0.009 | 0.051 | 0.009 | -0.156 | -0.044 |

**Table S5: PCA scores of Genotypes**

| **Genotypes** | **PC 1** | **PC 2** | **PC 3** | **PC 4** | **PC 5** | **PC 6** | **PC 7** | **PC 8** | **PC 9** | **PC 10** | **PC 11** | **PC 12** | **PC 13** | **PC 14** |
| --- | --- | --- | --- | --- | --- | --- | --- | --- | --- | --- | --- | --- | --- | --- |
| **GSF1** | 57.93 | 193.28 | -146.96 | 43.05 | -27.74 | 1.42 | 5.21 | -3.46 | -1.03 | -0.34 | 0.74 | -0.22 | 0.41 | -0.01 |
| **GSF2** | 946.65 | 149.26 | -157.75 | 58.31 | -28.36 | -24.73 | -2.26 | -0.57 | 1.54 | 0.48 | -2.04 | 0.22 | -0.04 | -0.03 |
| **GSF3** | 360.91 | 117.61 | 169.25 | -27.82 | 12.53 | -3.18 | 2.18 | -1.79 | -0.47 | 0.56 | -0.22 | -0.43 | -1.07 | 0.04 |
| **GSF4** | 273.61 | -79.23 | 172.16 | -34.52 | -40.13 | -19.59 | -3.61 | -1.47 | 0.59 | 0.40 | 1.99 | 0.69 | 0.01 | 0.03 |
| **GSF5** | -1170.90 | 17.70 | -67.10 | 35.93 | -22.75 | -10.33 | 1.69 | 4.21 | -2.05 | -0.75 | 0.05 | 0.98 | -0.26 | 0.08 |
| **GSF6** | -1842.60 | 115.77 | 94.34 | -41.29 | 36.13 | 3.97 | 0.23 | -1.25 | 1.56 | -2.43 | -0.44 | 0.79 | 0.15 | 0.18 |
| **GSF7** | -196.64 | 225.34 | -56.32 | 26.19 | 5.72 | 3.86 | 0.71 | 3.45 | 2.34 | 0.18 | 1.39 | -1.00 | -0.03 | -0.03 |
| **GSF8** | 696.95 | -23.79 | 341.91 | 32.74 | 12.86 | 2.17 | 2.47 | 1.02 | 0.08 | 0.61 | -0.59 | 0.42 | 0.37 | -0.41 |
| **GSF9** | -1086.80 | 23.49 | 136.74 | 40.92 | 22.62 | -2.43 | -1.40 | 0.15 | -1.05 | 2.21 | -0.40 | -0.46 | 0.42 | 0.40 |
| **GSF10** | -2192.00 | 67.08 | 12.76 | -54.98 | -23.47 | 11.77 | -4.43 | -0.22 | -1.44 | -0.92 | -0.52 | -1.02 | 0.05 | -0.25 |
| **YSF1** | 1240.30 | -310.04 | -17.19 | -133.67 | -14.16 | -15.60 | 3.04 | 1.10 | 0.32 | -0.59 | -0.61 | -0.77 | 0.21 | 0.12 |
| **YSF2** | 1157.80 | -237.94 | -95.25 | 111.82 | 49.85 | -16.91 | -2.04 | -0.72 | -0.80 | -1.44 | 0.60 | -0.46 | -0.06 | -0.08 |
| **YSF3** | -2230.30 | -207.82 | -276.04 | -46.63 | 25.64 | 4.97 | 0.88 | -0.79 | 0.63 | 2.00 | 0.21 | 0.59 | -0.11 | -0.19 |
| **YSF4** | 3257.10 | 208.62 | -131.05 | -89.47 | 25.12 | 19.95 | -2.33 | 0.59 | -1.07 | 0.29 | 0.16 | 0.57 | 0.12 | 0.01 |
| **YSF5** | 727.88 | -259.33 | 20.49 | 79.44 | -33.86 | 44.66 | -0.33 | -0.24 | 0.83 | -0.26 | -0.30 | 0.10 | -0.18 | 0.14 |
